# Differences in gut microbial composition correlate with regional brain volumes in irritable bowel syndrome

**DOI:** 10.1186/s40168-017-0260-z

**Published:** 2017-05-01

**Authors:** Jennifer S. Labus, Emily B. Hollister, Jonathan Jacobs, Kyleigh Kirbach, Numan Oezguen, Arpana Gupta, Jonathan Acosta, Ruth Ann Luna, Kjersti Aagaard, James Versalovic, Tor Savidge, Elaine Hsiao, Kirsten Tillisch, Emeran A. Mayer

**Affiliations:** 1 Division of Digestive Diseases, David Geffen School at UCLA, Los Angeles, CA 90095 USA; 2 Texas Children’s Microbiome Center, Department of Pathology, Texas Children’s Hospital, 1102 Bates Ave., Houston, TX USA; 3 Department of Pathology & Immunology, Baylor College of Medicine, One Baylor Plaza, Houston, TX USA; 4 Center for Human Nutrition, Washington University School of Medicine, St. Louis, MO 63110 USA; 5 Oppenheimer Center for Neurobiology of Stress and Resilience, CHS 42-210 MC737818 10833 Le Conte Avenue, Los Angeles, CA 90095-7378 USA

**Keywords:** Brain-gut-microbiome axis, Irritable bowel syndrome, Metagenome, *Firmicutes*, *Bacteroidetes*

## Abstract

**Background:**

Preclinical and clinical evidence supports the concept of bidirectional brain-gut microbiome interactions. We aimed to determine if subgroups of irritable bowel syndrome (IBS) subjects can be identified based on differences in gut microbial composition, and if there are correlations between gut microbial measures and structural brain signatures in IBS.

**Methods:**

Behavioral measures, stool samples, and structural brain images were collected from 29 adult IBS and 23 healthy control subjects (HCs). 16S ribosomal RNA (rRNA) gene sequencing was used to profile stool microbial communities, and various multivariate analysis approaches were used to quantitate microbial composition, abundance, and diversity. The metagenomic content of samples was inferred from 16S rRNA gene sequence data using Phylogenetic Investigation of Communities by Reconstruction of Unobserved States (PICRUSt). T1-weighted brain images were acquired on a Siemens Allegra 3T scanner, and morphological measures were computed for 165 brain regions.

**Results:**

Using unweighted Unifrac distances with hierarchical clustering on microbial data, samples were clustered into two IBS subgroups within the IBS population (IBS1 (*n* = 13) and HC-like IBS (*n* = 16)) and HCs (*n* = 23) (AUROC = 0.96, sensitivity 0.95, specificity 0.67). A Random Forest classifier provided further support for the differentiation of IBS1 and HC groups. Microbes belonging to the genera *Faecalibacterium*, *Blautia*, and *Bacteroides* contributed to this subclassification. Clinical features distinguishing the groups included a history of early life trauma and duration of symptoms (greater in IBS1), but not self-reported bowel habits, anxiety, depression, or medication use. Gut microbial composition correlated with structural measures of brain regions including sensory- and salience-related regions, and with a history of early life trauma.

**Conclusions:**

The results confirm previous reports of gut microbiome-based IBS subgroups and identify for the first time brain structural alterations associated with these subgroups. They provide preliminary evidence for the involvement of specific microbes and their predicted metabolites in these correlations.

**Electronic supplementary material:**

The online version of this article (doi:10.1186/s40168-017-0260-z) contains supplementary material, which is available to authorized users.

## Background

Alterations in gut microbial composition or “dysbiosis” have been implicated in the pathophysiology of irritable bowel syndrome (IBS) [1–3]. IBS is the most common chronic visceral pain syndrome. In the absence of agreed upon biomarkers, the syndrome is defined by symptom criteria which include the presence of chronically recurrent abdominal pain associated with alterations in bowel habits [4]. This hypothesis has been based on the reported findings of gut microbial differences between healthy control subjects (HCs) and IBS patients, on findings in rodent models of IBS, and on the theoretical appeal of such a hypothesis to explain various clinical observations. These include the observations in some patients that antibiotic treatment can both trigger and attenuate IBS symptoms [2], the development of IBS-like symptoms following gastroenteritis in a small percentage of patients [5], and the beneficial effects of the fermentable oligosaccharides, disaccharides, monosaccharides, and polyols (FODMAP) elimination diet and probiotic ingestion [2]. In addition, the well described interactions between the gut microbiota with the brain and the gut-based immune system provide plausible mechanisms consistent with a role of altered gut microbiota in IBS [6]. However, to date, few consistent differences have been identified in the gut microbiota between HCs and IBS. This may be in part related to the clinical (age, sex, medications) and physiological (intestinal transit, history of early adverse life events [EALs], stress reactivity) heterogeneity of patients selected solely by symptom criteria. One exception is an increased ratio between the phyla *Firmicutes* and *Bacteroidetes* (F-B ratio) which has been reported by several investigators [7–10]. A recent study provided evidence for subgroups of IBS patients based on gut microbial composition, which were independent of predominant bowel habits and established symptoms criteria [10].

There is a growing consensus that alterations in the bidirectional interactions of the central nervous system with the gut (brain-gut axis) play an important role in IBS pathophysiology (reviewed in [6]). In animal models, early adverse life events have been shown to be associated with stress-induced alterations in intestinal transit (indexed by increased fecal pellet output, [11] and in the composition of the gut microbiota [12, 13]. In addition to a growing list of abnormalities described by different investigators in the different components of the peripheral gut connectome [14, 15], both structural and functional alterations of the brain have been identified in IBS subjects [16]. Recent analyses have identified widespread gray and white matter alterations in the brain of IBS patients, including extensive changes in the somatosensory system (thalamus, basal ganglia, S1, and M1). A history of early adversity has also been shown to be correlated with brain structural changes [17]. A recent study showed cross sectional correlations between brain structure and gut microbial composition [18]. The fact that functional activity in some of the same regions (somatosensory regions, basal ganglia) was increased when healthy women consumed a probiotic mix over 4 weeks [19] suggests the possibility of a link between gut microbial composition and sensory processing and integration in the brain, both in healthy control subjects (HCs) and in a subgroup of IBS patients.

By studying a group of well phenotyped IBS subjects and HCs, we aimed to address three questions related to gut microbiota, clinical parameters, and brain signatures: 1. Can gut microbial composition be used to classify IBS patients and to identify subgroups with different clinical and behavioral symptoms, as recently suggested? 2. Is there an association between a history of early adverse life events (EALs) and gut microbial composition? 3. Is there a relationship between gut microbial composition and IBS-related brain biomarkers? Based on past clinical literature and brain regions altered in IBS, we expected to see a relationship between microbiota and emotion-related (amygdala, hippocampus) and sensory brain regions (basal ganglia, posterior insula, and primary sensory and motor regions).

## Methods

### Participants

Stool samples were collected at UCLA from 29 right-handed adult IBS patients (22 females) and 23 HCs (14 females), who also underwent multimodal brain imaging studies at UCLA. IBS subjects met Rome III symptom criteria for IBS [20]. A gastroenterologist or nurse practitioner obtained a medical history and physical exam to confirm the IBS diagnosis. IBS patients with any bowel habit were included. With regard to IBS diagnostic subtypes, 11 subjects had IBS-constipation, 10 subjects had IBS-diarrhea, and 8 subjects had either alternating (1), mixed (5), or unspecified (2) bowel habits. The Mini-International Neuropsychiatric Interview was used to identify past or current psychiatric illness [21].

Exclusionary criteria for all subjects included (1) serious medical conditions or were taking medications which could compromise interpretation of the brain imaging; (2) ongoing major psychiatric diagnoses or use of psychotropic medications in the past 6 months (subjects were not excluded for lifetime incidence of psychiatric disorders or for intake of low-dose tricyclic antidepressants for non-psychiatric indications); (3) use of antibiotics in the past 3 months, selective serotonin reuptake inhibitors, opioids; and (4) excessive physical exercise (e.g., marathon runners). All procedures complied with the principles of the Declaration of Helsinki and were approved by the Institutional Review Board at our institution. All authors had access to the study data and reviewed and approved the final manuscript.

### Questionnaires

The Hospital Anxiety and Depression Scale [HADs] [22] and the Patient Health Questionnaire-15 [PHQ] were obtained to assess mood [23]. The Early Traumatic Inventory–Self Report (ETI-SR) [24] was used to access histories of childhood traumatic and adverse life events that occurred before the age of 18 years old and covers four domains: general trauma (31 items), physical (9 items), emotional (7 items), and sexual abuse (15 items) [24]. The Catastrophizing subscale from the Coping Strategies Questionnaire was administered to assess levels of catastrophizing [25]. The degree to which subject viewed situations as stressful in the past month was measured by the Perceived Stress Scale [26]. Medication usage was defined by endorsement of any of the following: antispasmodic, laxatives, stool_softener, fiber supplement, nonsteroidal anti-inflammatory drugs, aspirin, acetaminophen, thyroid medications, antihistamine, or proton pump inhibitors.

A 1-month qualitative Food Frequency Questionnaire (FFQ) from the third National Health and Nutrition Examination Survey (NHANES III)^1^ was used to collect information on participants’ eating patterns. The FFQ data was processed using the National Cancer Institute’s Diet*Calc software (Diet*Calc Analysis Program, Version 1.4.3. National Cancer Institute, Applied Research Program. November 2005.), which provides estimates of energy and nutrient intake [27]. A participant’s total animal fat intake was calculated by summing the total fat (in grams) of foods containing animal sources of fat (Additional file 1: Table S1). Combination foods such as soups and casseroles were excluded due to inability to differentiate animal and plant fat sources. Using SPSS (IBM Corp. Released 2013. IBM SPSS Statistics for Windows, Version 22.0. Armonk, NY: IBM Corp.), the mean intake of animal fat was calculated for each group and compared across the three groups using univariate analysis of variance.

### Neuroimaging, segmentation, and parcellation

T1-weighted images were acquired on a Siemens Allegra 3 Tesla scanner, repetition time = 2200 ms, echo time = 2.85 s, inversion time = 750 ms, flip angle = 20°, field of view = 220 × 220 mm, resolution = 256 × 256, slices per volume = 176, slice thickness = 1 mm, voxel size = 0.86 × 0.86 × 1 mm. T1-image segmentation and regional parcellation were conducted using FreeSurfer [28–30] following the nomenclature described in the Destrieux and Harvard-Oxford subcortical atlas [31, 32]. For each cerebral hemisphere, a set of 74 cortical structures were labeled in addition to 7 subcortical structures and to the cerebellum. One additional midline structure (the brain stem) was also included, for a complete set of 165 parcellations for the entire brain. Four representative but distinct morphological measures were computed for each cortical parcellation: gray matter volume, surface area, cortical thickness, and mean curvature. A list of all the regions is provided in Additional file 2: Table S2.

### Microbial analysis

#### Intestinal microbial composition: stool collection, processing, and analysis of 16S rRNA gene sequencing data

Stool specimens were stored in an RNA stabilizing reagent (RNALater) at −80 °C following collection at UCLA. They were later placed in cryo-containers and shipped to the Texas Children’s Microbiome Center. Bacterial DNA from the self-collected stool specimens was extracted, amplified, and sequenced, as previously described.[9, 33–35] Briefly, 16S ribosomal RNA (rRNA) gene sequence libraries were generated using the V3-V5 (357F/926R) primer region [9, 36] and sequenced on the 454 platform (Life Sciences, Branford, CT, USA). The sequence libraries were parsed by barcode and quality filtered using the Genboree Microbiome Toolset [35], where sequences shorter than 200 bp, having average quality scores <20, containing ambiguous base calls, or including mismatches to barcode or sequencing primer were culled. After quality filtering and the removal of barcode and primer sequences, all remaining sequences were clustered into operational taxonomic units (OTUs) at a 97% similarity level using QIIME (Quantitative Insights into Microbial Ecology, v1.3.0) [37]. OTUs were clustered using the CD-Hit algorithm [38], and reads were screened for chimeras using ChimeraSlayer [39]. All potential chimeras were excluded from further analysis. OTU identities were assigned using the Ribosomal Database Project Classifier [40] with the Greengenes reference database (version 12-10) and confidence scores ≥80%. To accommodate for variance in sequencing depth, all sequence libraries were randomly sub-sampled to a depth of 1355 sequences, the size of the smallest quality filtered sequence library, prior to downstream analysis. Bacterial community profiles were analyzed by global parameters (described below) including determination of diversity, evenness, richness, and relative abundance of the bacteria identified in each sample. Output sequences were classified at the domain, phylum, family, genus, and species levels where possible, depending on the depth of reliable classifier assignments [41].

#### Diversity analysis

Alpha-diversity metrics (i.e., bacterial diversity within a sample) were computed and included Faith’s phylogenetic diversity metric, chao1 richness estimator, Shannon’s entropy, and counts of observed OTUs [42, 43]. Between-group differences in alpha diversity were evaluated using a nonparametric *t* test, and 1000 Monte Carlo permutations were used to calculate the nonparametric *p* value. In addition, for each group, alpha-diversity curves were generated for each diversity metric at different rarefaction depths.

#### Determining relationships among samples

A phylogenetically informed distance matrix was computed using the unweighted UniFrac metric [44]. Hierarchical clustering using average linkage was performed to visualize relationships among the samples based on similarity of microbial composition. As recommended by Navas-Molina et al. [42], principal coordinate analysis (PCoA) was used to evaluate the presence of clusters or groupings in the data. PCoA provides information regarding the largest source of variation in the data and allows the observation of similarities and differences between samples. Pearson correlations of OTU relative abundance versus principal coordinate axis scores were used to identify taxa contributing to the separation of samples in PCoA space. PCoA indicated the presence of three groups having similar microbial profiles, and these are labeled as HCs, HC-like IBS, and IBS1 throughout the manuscript.

Adonis analysis, a permutational analysis of variance, was performed to test for differences between groups in overall microbial composition [45].

Random forest analysis was used to evaluate the degree to which fecal communities could classify group and identify features differentiating IBS microbial communities from controls. The caret package (version 6.0-73) for *R* [46] was used with repeated cross-validation (10-fold, five repeats). Receiver-operating characteristic (ROC) curves and area under the ROC (AUROC) were calculated using the pROC package for R (version 1.8) [47]. Any OTU occurring in fewer than 10% of subjects was filtered prior to implementing the random forest algorithm.

#### Group differences in taxonomic abundance and clinical metadata

Group differences in relative abundance at each taxonomic level (phylum, class, order, family, genus, and OTUs/“species”) and the ratio of *Firmicutes* to *Bacteroidetes* were tested using the nonparametric Kruskal Wallis test correcting for multiple comparisons with the Benjamini-Hochberg false discovery rate (FDR) procedure and confirmed using pairwise comparisons. FDR correct *p* values (*q*) are reported. Independent sample *t* tests were used to test for group differences in groups on symptom severity, mood, and emotional trauma scores.

#### Association between brain structure and microbiota

Using Matlab, partial correlational analysis controlling for total gray matter volume was used to examine the association between the relative abundance of the phyla *Bacteroidetes* and *Firmicutes* showing group differences and all 165 brain volumes in IBS (*n* = 29). We also tested for differences in the brain morphometry of somatosensory-, motor-, and emotional-related regions between the microbiota-derived clusters, IBS1, HC-like IBS, and HC using contrast analysis within the framework of the general linear model. Group was specified as a factor, and total gray matter volume was specified as a covariate. Somatosensory and motor regions included the basal ganglia (putamen, caudate nucleus, nucleus accumbens, globus pallidus), primary sensory (postcentral gyrus, postcentral sulcus, central sulcus), secondary sensory (subcentral gyrus (central operculum) and sulci), motor (precentral gyrus, inferior part of the precentral sulcus, superior part of the precentral sulcus), and mid and posterior insula (long insular gyrus and central sulcus of the insula, inferior segment of the circular sulcus of the insula, posterior ramus of the lateral sulcus) regions. Emotional-related regions included the amygdala and the hippocampus. Analyses examining the relationship between brain structure and microbiota analyses were considered exploratory and important for future hypotheses generation. We report uncorrected *p* values as Type II error control and is equally important in exploratory research. We caution against reliance on significance testing and instead emphasize interpretation of effect sizes of brain-microbiota associations for future studies.

#### Predicted metagenomics analysis

The metagenomic content of samples was inferred from 16S rRNA gene sequence data using PICRUSt 1.0 and the KEGG database, which includes 6909 bacterial genes (i.e., metagenes) annotated in reference genomes [48]. The data were filtered to remove metagenes present in less than five samples or with an overall abundance less than 10^−6^. Group differences in predicted metagenes were identified using DESeq2 in *R*, which employs an empirical Bayesian approach to shrink dispersion and fit non-rarified count data to a negative binomial model [49]. *P* values were adjusted for multiple hypothesis testing using the Benjamini-Hochberg FDR procedure. DESeq2 was also employed to identify metagenes with a statistically significant association with brain structural features that differed among the three microbial clusters. Selected metagenes associated with brain morphometry and microbiome clusters were further analyzed with FishTaco (http://borenstein-lab.github.io/fishtaco) [50]. This method uses a permutation approach and Shapley value analysis to quantitate the contribution of individual taxa to shifts in metagene abundance between two clusters (https://borenstein-lab.github.io/fishtaco/). Bioinformatics is available as Additional file 3.

## Results

### Clinical characteristics

The means and standard deviation for descriptive data describing the samples can be found in Table 1. The average ages for IBS (*n* = 29, females = 21) and HCs (*n* = 23, females = 14) were similar, 26.1 years (SD = ±5.72 and 26.0 years (±6.5), respectively. Average symptom duration in IBS subjects was 11.3 (±13.2) years. Even though IBS subjects as a group had significantly greater level of anxiety (*p* = .03) but not depression symptoms, the anxiety levels for the majority of subjects were within the normal range. However, six IBS subjects (HAD score range 12–17) and one HC (HAD score 13) had scores greater than 11, indicating a probable level of clinical anxiety. Compared to HCs, IBS reported greater levels of perceived stress (*p* = .02) and higher levels of catastrophizing (*p* = .02). No group differences were observed in total emotional trauma history scores, but there was a trend for a greater history of emotional trauma in IBS (*p* = .09). Moderate to high levels of early life trauma (4–5) on the emotional subscale were reported by 36% IBS subjects while no healthy control subjects showed such scores. IBS subjects reported average of overall symptoms in the past week of 9.3 (±4.3) on a 21-point numeric rating scale (0 = no pain, 20 = the most intense symptoms imaginable). Distribution of bowel habit was 11 constipation (37.9%), 10 diarrhea (34.5%), 1 alternating (3.4%), 5 mixed (17.2%), and 2 unspecified (6.9%].

**Table 1:** Group demographic and clinical characteristics^a^

	HC			IBS				
	M	SD	*N*	M	SD	*N*	*t*	*p*
Sex (% female)^b^	61%		23	72%		29		.55
Age	26.00	6.48	23	26.07	5.72	29	−0.04	.97
Body mass index	22.79	8.54	23	23.18	6.44	29	−0.18	.86
HAD anxiety	4.61	3.31	23	7.34	5.15	29	−2.21	.03
HAD depression	2.00	2.02	23	3.24	3.09	29	−1.66	.10
ETI_General_Score^c^	1.57 (0–6)	1.53	23	2.00 (0–5)	1.69	27	−0.95	.35
ETI_Physical_Score	1.50 (0–5)	1.79	22	1.33 (0–4)	1.57	27	0.35	.73
ETI_Emotional_Score	.41 (0–1)	.908	22	1.19 (0–5)	1.90	27	−1.76	.09
ETI_Sexual_Score	.26 (0–4)	.915	23	.42 (0–5)	1.24	26	−0.52	.61
ETI_Total_Score	3.82 (0–15)	3.61	22	4.69 (0–17)	4.82	26	−.70	.49
Perceived stress score	12.73	7.04	22	17.39	7.08	28	−2.32	.02
Coping Scale Questionnaire	.67	1.08	21	1.56	1.44	27	−2.34	.02

*Abbreviations*: *HC* healthy controls, *IBS* irritable bowel syndrome, *M* mean, *P* probability, *SD* standard deviation, *t* independent t statistic

^a^Clinical characteristics for healthy controls and subjects with irritable bowel syndrome

^b^Percentage of female in each group, *p* value from Fisher’s exact test

^c^Range of scores in parentheses

No statistically significant differences in dietary patterns were observed between groups. IBS1 showed slightly higher BMI than HC-like IBS (*p* = .047) although both groups had an average body weight within the normal range.

### IBS subgroup identified based on a microbiome signature

**Fig. 1:**
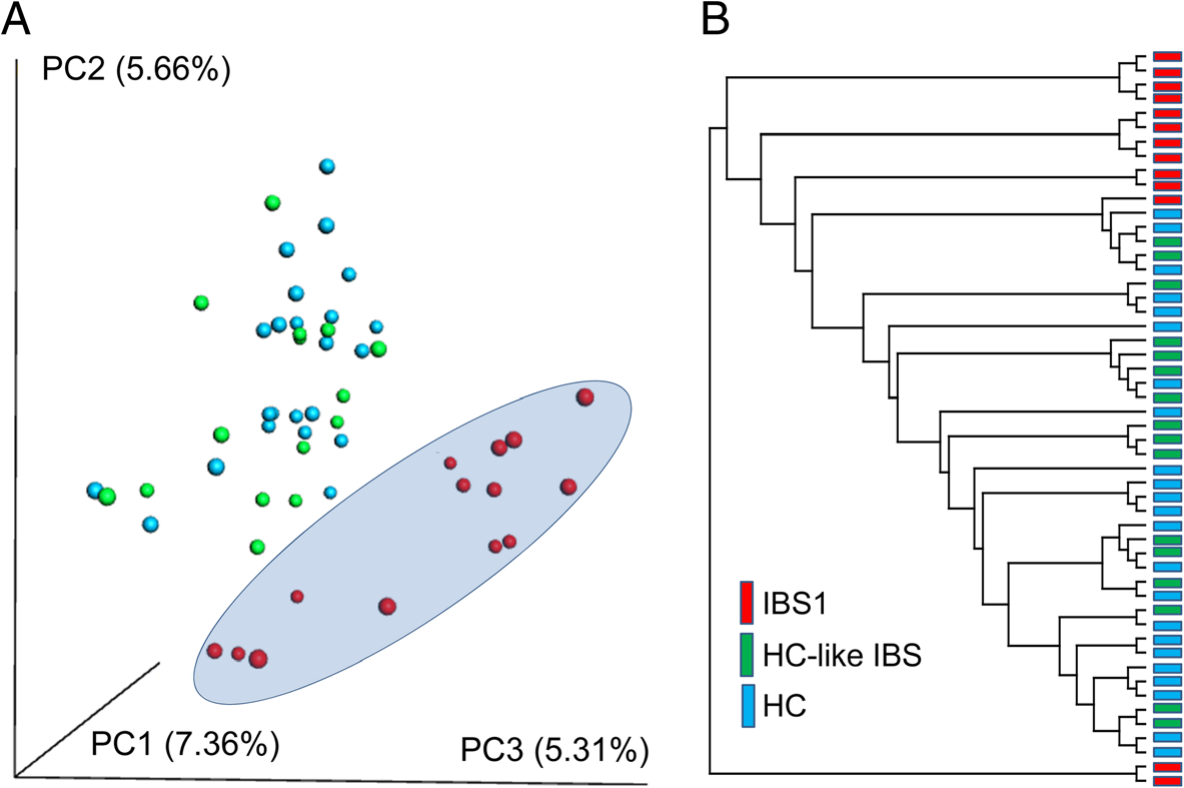
16s rRNA gene data revealed two distinct IBS subgroups, one indistinguishable from healthy controls. **a** Principal coordinate analysis (PCoA) on the unweighted Unifrac distance matrix from the rarefied data was used to evaluate the presence of clusters or groupings based upon operational taxonomic unit (OTU)-level microbial features. **b** Hierarchical clustering using average linkage was performed to visualize relationships among the samples based on similarity of microbial composition. Both procedures operate on a phylogenetically informed distance matrix computed using the unweighted UniFrac metric

Hierarchical clustering using average linkage and PCoA analysis on unweighted Unifrac distances are depicted in Fig. 1a, b and Additional file 4: Figure S1. Together, these analyses indicated that microbial signatures could be used to group patients and to discriminate among samples. Based on the PCOA and hierarchical clustering, the samples were labeled as IBS1 (IBS patients with microbial profiles distinct from HCs; *n* = 13), HC-like IBS (IBS patients with similar microbial composition as HCs; *n* = 16) and HCs (*n* = 23). One IBS subject clustered with IBS1 in the PCoA but clustered with HCs in the hierarchical clustering. This subject was labeled as HC-like IBS subject. We tested whether this group assignment changed group difference or correlation results but it did not. Adonis analysis [45] of the unweighted Unifrac distances indicated that a significant proportion of the variance could be explained by IBS1–HC differences *F*(1,51) = 5.79, *p* = .004, *R* ^2^ = .10. The variance accounted for other group contrasts was not statistically significant (HC-like IBS-IBS1, *F*(1,51) = 2.2, *p* = .073, *R* ^2^ = .04; HC-like IBS-HCs, *F*(1,51) = 1.23, *p* = .25, *R* ^2^ = .02).

OTUs contributing to the variation in microbial signatures along the 3rd axis of the PCoA plot (i.e., that which captured the separation of the IBS1 communities from the HC and HC-like IBS communities) were identified using correlation analysis. Those whose relative abundances correlated most strongly with the location along this axis are provided in Additional file 5: Table S3 and included members of the genera *Faecalibacterium*, *Bacteroides*, and *Blautia*.

Random forest analysis provided further support for the differentiation of IBS1 and HC groups. This analysis identified an OTU-based signature which correctly distinguished the majority of IBS1 subjects from HC subjects. Using 10-fold cross-validation, the resulting model had an AUROC of 0.96, sensitivity of 0.95, and specificity of 0.67 (Additional file 6: Figure S2). OTUs contributing to the differentiation of IBS1 and HC gut communities, according to their random forest importance (i.e., mean decrease in accuracy) scores, included members of the genera *Blautia*, *Streptococcus*, *Faecalibacterium*, and *Bacteroides* (see Additional file 7: Table S4).

### Diversity analyses and comparison of bacterial relative abundances among IBS subgroups

Alpha diversity by groups (HC, IBS) and subgroups (IBS1, HC-like IBS, HC) were calculated, and results are depicted in Additional file 8: Figure S3. Comparison of Faith’s phylogenetic diversity indices with nonparametric *t* tests indicated that as a group, all IBS subjects (*n* = 29) showed significantly greater diversity than HCs, *t*(50) = 2.83, *p* = .007. Examining the IBS subgroups indicated this was largely due to the fact that the IBS1 community was more diverse than the HC-like IBS patients, *t*(27) = 3.33, *p* = .012, and HCs, *t*(36) = 3.80, *p* = .003. There were no differences observed between the HC-like IBS subjects and HCs, *t* = .90, *p* = .99. Similar results were observed for the other diversity measures: chao1 richness estimator, Shannon’s entropy, and number of observed OTUs.

**Fig. 2:**
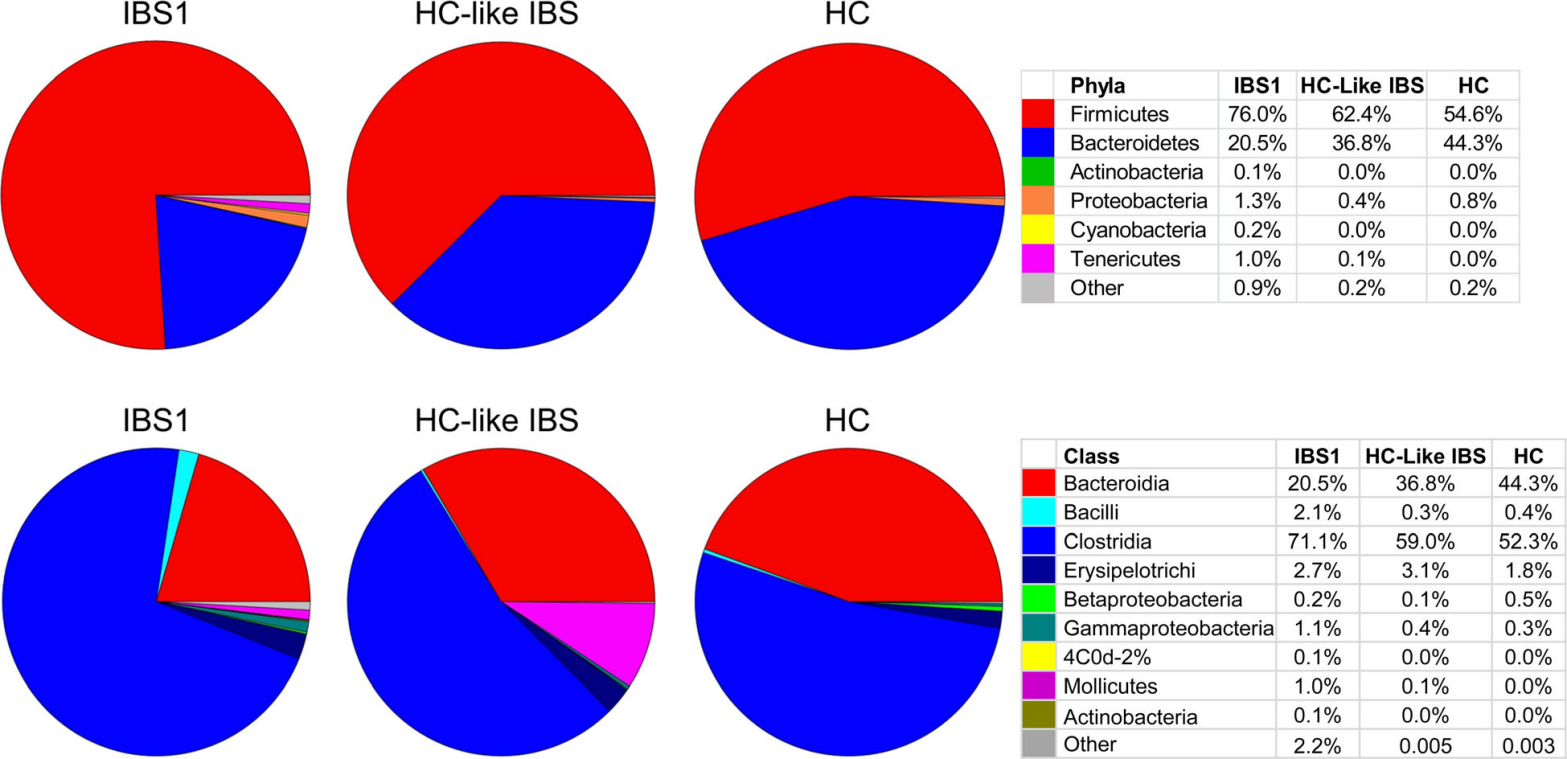
Microbial composition of each group at the phylum and class level. Pie charts show the proportion of reads in each phylum (*top*) and class (*bottom*) for IBS1, HC-like IBS, and HCs

**Fig. 3:**
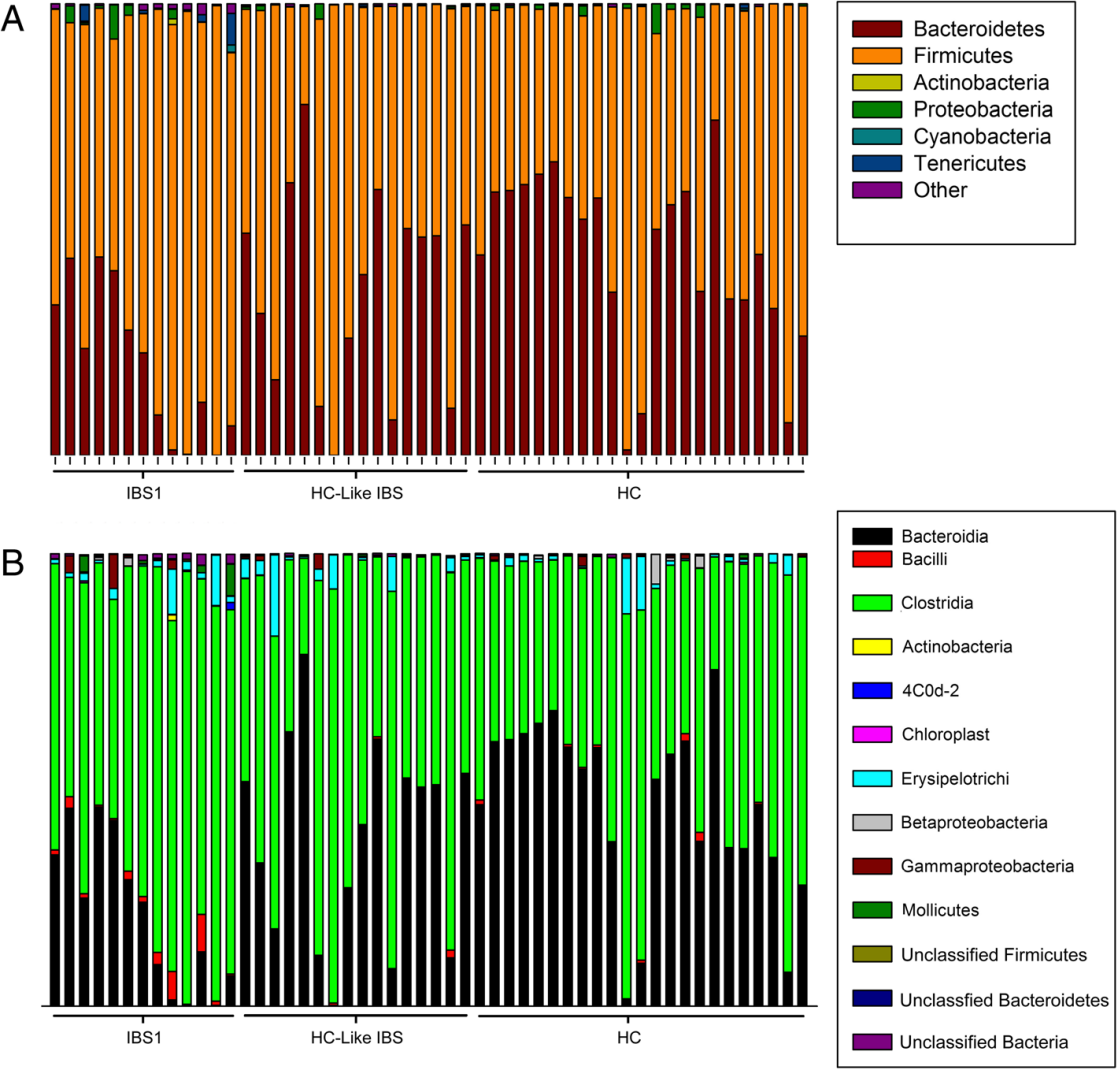
Microbial composition of each sample by group at the phylum and class level. *Stacked vertical bar charts* depict the variability in phyla- and class-level composition for individuals by groups

**Fig. 4:**
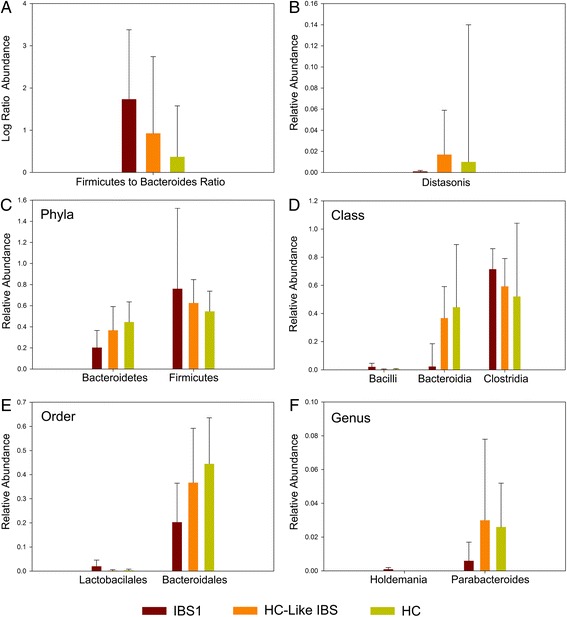
Group differences in the relative abundance of microbiota. *Bar graphs* depict the *Firmicutes* to *Bacteroidetes* ratio (**a**), the mean relative abundance for identifiable operational taxonomic units (**b**), and taxa demonstrating group differences at each taxonomic level (phylum, class, order, family, and genus (**c**-**f**)). *Error bars* represent standard deviations

Figures 2 and 3 depict the microbial composition of each group and individual at the phylum and class levels. The ratio of *Firmicutes* to *Bacteroidetes* (F-B ratio) was significantly higher in the IBS1 subgroup compared to HCs (*q* = .02). The F-B ratio in the HC-like IBS group did not differ significantly from either group (Fig. 4a).

The relative abundances of OTUs and the mean relative abundance for identifiable taxa demonstrating overall group differences at each taxonomic level (phylum, class, order, family, and genus) are depicted in Fig. 4. The means and standard deviations as well as Kruskall Wallis tests of significance can be found for identifiable and unidentified taxa and OTUs in Additional file 9: Table S5.

At the phylum level (Fig. 4c), the relative abundance of *Firmicutes* was significantly greater in the IBS1 subjects compared to HCs (*q* = .002) but not the HC-like IBS subjects (*q* = .075). On the other hand, the relative abundance of *Bacteroidetes* was significantly lower in IBS1 group compared to HCs (*q* = .003) but not in the HC-like IBS subjects (*q* = .12). For all comparisons, the HC-like IBS group did not differ from the HCs with respect to the relative abundance of either *Firmicutes* or *Bacteroidetes*. Furthermore, no differences were observed at the phylum level for the relative abundance of Actinobacteria (IBS1 .1%, HC-like IBS 0%, and HCs 0%) or Proteobacteria (1.3, .8, .4%).

At the class level (Fig. 4d), differences were observed in the *Firmicutes* classes, *Bacilli* and *Clostridia*. The relative abundance of members of *Bacilli* was greater in IBS1 compared to HC-like IBS (*q* = .001) and HCs (*q* = .005). For *Clostridia*, IBS1 showed greater abundances than HCs (*q* = .004) but not HC-like IBS (*q* = .14). IBS1 had lower abundances of *Bacteroidetes* class *Bacteroidia* compared to HCs (*q* = .002) but not HC-like IBS (*q* = .075).

At the order level (Fig. 4e), differences were observed with respect to the *Bacilli* order *Lactobacillales* with greater abundance observed for IBS1 compared to HC-Like IBS (*q* = .004) and HCs (*q* = .004). The relative abundance of *Bacteroidales* order was lower in IBS1 compared to HCs (*q* = .002) but not HC-like IBS (*q* = .075).

At the genus level (Fig. 4f), members of the Erysipelotrichaceae genus *Holdemania* were more abundant among IBS1 compared to HC-like IBS (*q* < .001) and HCs (*q* < .001). Members of the Porphyromonadaceae genus *Parabacteroides* were less abundant in IBS1 compared to HCs (*q* = .003) but not HC-like IBS (*q* = .097).

### Mean differences in clinical metadata between IBS subgroups based on microbial clusters

Detailed clinical data for the subgroups of IBS based on microbial community profiles, IBS1 (9 females, 5 males) and HC-like IBS (12 females, 3 males), are available in Additional file 10: Table S6. IBS1 subjects reported a longer duration of symptoms than HC-like IBS (*t*(26) = 2.80, *p* = .01) as well as higher scores on the ETI emotional subscale, *t*(25) = 3.14, *p* = .004. Although overweight status (BMI >25.0) was not significantly different between the two IBS clusters, IBS1 [m(sd), 25.52 (5.68)] showed a trend (*t*(27) = 1,99, *p* = .057) toward greater BMI compared to HC-like IBS [20.99 (6.51)], Hedge’s effect size *g* (Cohen’s *d* corrected for small sample sizes) = .74. This was associated with a trend for higher dietary plant fat intake in the IBS1 group compared to HC-like IBS (*p* = .047) and HC (*p* = .017).

In contrast, microbial community clustering in IBS was not associated with sequencing depth, age, predominant bowel habit, symptoms of anxiety or depression, levels of catastrophizing, perceived stress, or medication usage.

Subgroup analysis in only females indicated that the mean differences in the duration of symptoms, *t*(19) = 2.72, *p* = .014, and emotional ETI scores remained significant as did the trend for greater BMI (*t*(19) 1.80, *p* = .08 in IBS.

### Associations between clinical metadata and microbiota taxa differentiating IBS1 from HC-like IBS and HCs

#### Order level taxa

In IBS subjects, *Lactobacillales*, which had significantly higher abundance in IBS1, had a moderately positive correlation with ETI total score (*r* = .51, *p* = .008), as well as sexual (*r* = .59, *p* = .001), physical (*r* = .51, *p* = .007), and emotional (*r* = .42, *p* = .028) subscores. No correlations were observed for *Bacteroidales*. Examining correlations in female IBS only revealed that *Bacteroidales* positively correlated with overall symptom severity (*r* = .423, *p* = .056). Correlations with the ETI total score (*r* = .56, *p* = .01), and its physical (*r* = .53, *p* = .013), emotional (*r* = .52, *p* = .02), and sexual (*r* = .58, *p* = .008) subscores persisted.

#### Genus level taxa

Abundance of *Parabacteroides* was positively associated with bloating *r* = .48 *p* = .03. Because *Holdemania* was not identified in any group but IBS1, associations with this genus were not examined.

Across all subjects (*n* = 48), the relative abundance of the *Firmicutes*-associated class *Bacilli* was positively correlated with ETI total score (*r* = .34, *p* = .018) as well as with sexual (*r* = .43, *p* = .002) and emotional (*r* = .38, *p* = .007) subscores. No correlations with EALs were found for the *Firmicutes*-associated class *Clostridia* or the *Bacteroidetes*-associated class *Bacteroidia*. IBS1 had greater scores on the emotional scale of the ETI than HC-like IBS (*p* = .004) and HCs (*p* = .001), while no differences were observed between IBS and HCs as a group for this score.

### Association between brain structure and discriminative microbiota in IBS subjects

Table 2 shows the results of the correlations between the relative abundance of classes of bacteria associated with *Firmicutes* and *Bacteroidetes* that discriminated the microbial subgroups (Fig. 4d) and the parcellated 165 regional brain volumes for all IBS (*n* = 29). Moderately sized correlations were observed for the *Clostridia* belonging to phylum *Firmicutes* (higher in IBS1), and for the *Bacteroidia* belonging to phylum *Bacteroidetes* (lower in IBS1) with several sensory integration regions including the thalamus, basal ganglia (caudate nucleus, putamen, pallidum, nucleus accumbens), and the superior part of the precentral gyrus (motor cortex). Similar correlations were found for the anterior insula and ventral prefrontal regions.

**Table 2:** Partial correlations between class-level taxa and brain regions controlling for total gray matter volume in IBS (*N* = 29)

Brain region	Firmicutes-associated Bacilli		Firmicutes-associated Clostridia		Bacteroidetes-associated Bacteroidia	
	r	p	r	p	r	p
Subcortical						
L thalamus	−.15	.461	**−.40**	**.037**	.36	.063
L caudate nucleus	.28	.156	**.49**	**.010**	**−.45**	**.017**
L putamen	.18	.376	**.52**	**.006**	**−.53**	**.005**
R putamen	.11	.600	**.42**	**.029**	**−.48**	**.012**
L pallidum	.30	.132	.37	.056	**−.44**	**.023**
R pallidum	.35	.070	.36	.069	**−.45**	**.019**
L NACC	.20	.326	.38	.052	**−.45**	**.019**
R NACC	**.42**	**.029**	**.53**	**.004**	**−.59**	**.001**
R CeB	−.20	.306	−.34	.080	**.41**	**.032**
Insula						
Horizontal ramus of the anterior segment of the lateral sulcus	.15	.462	**−.42**	**.027**	.35	.069
R short insular gyrus	−.12	.535	**−.51**	**.007**	**.44**	**.023**
Vertical ramus of the anterior segment of the lateral sulcus	−.20	.325	**−.48**	**.012**	**.49**	**.009**
Frontal						
R Orbital part of the inferior frontal gyrus	**−.43**	**.025**	**−.43**	**.027**	**.49**	**.009**
R orbital gyrus	**−.44**	**.020**	**−.39**	**.043**	**.47**	**.013**
R gyrus rectus	−.34	.083	**−.47**	**.014**	**.46**	**.015**
R superior part of the precentral gyrus	−.23	.252	**−.42**	**.031**	**.41**	**.033**
L triangular part of the inferior frontal gyrus	**.46**	**.017**	−.10	.604	.05	.790
Cingulate						
Posterior-ventral part of the cingulate gyrus	**−.39**	**.043**	−.11	.580	.07	.716
Parietal						
R supramarginal gyrus	**.50**	**.008**	.27	.167	−.28	.159
L_parietal occipital sulci	.01	.969	**−.50**	**.008**	**.45**	**.018**
Temporal						
L temporal pole	**−.39**	**.046**	−.21	.284	.24	.228
L planum polare of the superior temporal gyrus	−.07	.747	**.41**	**.035**	−.37	.060
L lateral occipital temporal sulcus	−.02	.924	**.45**	**.018**	**−.44**	**.021**
Occipital						
L superior and transvers occipital sulci	.28	.153	**.39**	**.043**	**−.45**	**.017**

Pearson’s correlation between relative abundance and brain volumes

R=correlation, p=probability

P values less than .05 uncorrected are bolded

Based on the relative abundance of these classes of bacteria, volumes in the sensory brain regions were increased, while volumes of insula and prefrontal cortices were decreased in IBS1 compared to HC-like IBS. Very few correlations were observed for the parietal, occipital, and temporal regions. The abundance of the *Bacilli* belonging to phylum *Firmicutes* showed correlations with the volume of fewer regions including the nucleus accumbens, prefrontal cortices, and ventral posterior cingulate cortex.

As can be seen in Table 3, compared to HC-like IBS and HCs, IBS1 had smaller cortical thickness and larger surface area in the anterior insula. Compared to HC-like IBS, IBS1 also showed greater volume and surface area in the posterior insula, right globus pallidum, and lower cortical thickness and surface area of motor cortex. Finally, compared to HCs, IBS has greater volume in the mid/posterior insula.

**Table 3:** Brain morphometry differences based on microbiota profiles

IBS 1-HC-like IBS					
Brain area	Region of interest	Brain measure	*p* value	β value	Standard error
Somatosensory network					
pINS	Left posterior ramus of the lateral sulcus	SA	.002	60.73	18.47
pINS	Right inferior segment of the circular sulcus of the insula	SA	.005	41.00	13.89
pINS	Right inferior segment of the circular sulcus of the insula	V	.005	120.31	41.20
pINS	Left posterior ramus of the lateral sulcus	V	.015	117.42	46.39
pINS	Right long insular gyrus and central sulcus of the insula	MC	.045	−0.01	0.01
Basal ganglia	Right globus pallidus	V	.007	102.28	36.47
Motor	Left superior part of the precentral sulcus	CT	.009	−0.06	0.02
Motor	Right superior part of the precentral sulcus	V	.006	−246.50	84.96
Motor	Right superior part of the precentral sulcus	SA	.017	−96.24	39.02
Motor	Right superior part of the precentral sulcus	CT	.026	−0.06	0.03
Motor	Right subcentral gyrus (central operculum) and sulci	CT	.020	−0.08	0.03
aINS	Left anterior segment of the circular sulcus of the insula	CT	.024	−0.09	0.04
aINS	Right anterior segment of the circular sulcus of the insula	CT	.029	−0.13	0.06
aINS	Right anterior segment of the circular sulcus of the insula	SA	.004	45.65	15.12
aINS	Right anterior segment of the circular sulcus of the insula	V	.044	79.04	38.16
IBS1 - HC					
pINS	Left long insular gyrus and central sulcus of the insula	V	.009	81.33	30.10
pINS	Left long insular gyrus and central sulcus of the insula	SA	.011	20.87	7.93
pINS	Right superior segment of the circular sulcus of the insula	CT	.045	−0.05	0.02
pINS	Left superior segment of the circular sulcus of the insula	CT	.042	−0.05	0.02
aINS	Right anterior segment of the circular sulcus of the insula	CT	.003	−0.16	0.05
aINS	Right anterior segment of the circular sulcus of the insula	SA	.002	43.67	13.51
Somatomotor	Left central sulcus	CT	.043	0.04	0.02
Somatomotor	Right central sulcus	CT	.033	0.04	0.02
HC-like IBS - HC					
Somatosensory	Left postcentral sulcus	CT	.005	0.06	0.02
Somatosensory	Right postcentral sulcus	CT	.011	0.05	0.02
Somatomotor	Right central sulcus	CT	.009	0.05	0.02
Somatomotor	Left central sulcus	CT	.018	0.04	0.02
pINS	Right inferior segment of the circular sulcus of the insula	V	.001	−122.97	35.48
pINS	Right inferior segment of the circular sulcus of the insula	SA	.031	−28.23	12.75
pINS	Right inferior segment of the circular sulcus of the insula	CT	.022	−0.07	0.03
pINS	Left posterior insula gyrus	CT	.037	0.08	0.04
pINS	Left long insular gyrus and central sulcus of the insula	MC	.035	0.01	0.01
pINS	Right long insular gyrus and central sulcus of the insula	MC	.011	0.01	0.01
pINS	Left posterior ramus of the lateral sulcus	SA	.034	−37.78	17.27
Basal ganglia	Right nucleus accumbens	V	.029	−39.23	17.45

This table contains results of the contrast analysis within the framework of the general linear model. Contrast were coded based on the three level cluster factor; IBS1-HC-like IBS (1 -1 0), IBS1-HC (1 0 -1), HC-like IBS-HC (0 1 -1). As such positive beta values indicate greater values in the first group listed. Negative values reflect greater value in the second group

*Abbreviations*: *INS* anterior insula, *pINS* posterior insula, *CT* cortical thickness, *SA* surface area, *V* volume, *MC* mean curvature

Compared to HCs, HC-like IBS had greater cortical thickness in the sensory brain regions, including the left post central and central sulci and the right posterior insula.

### Metagenes enriched in the IBS1 subgroup are associated with posterior insula morphometry

**Fig. 5:**
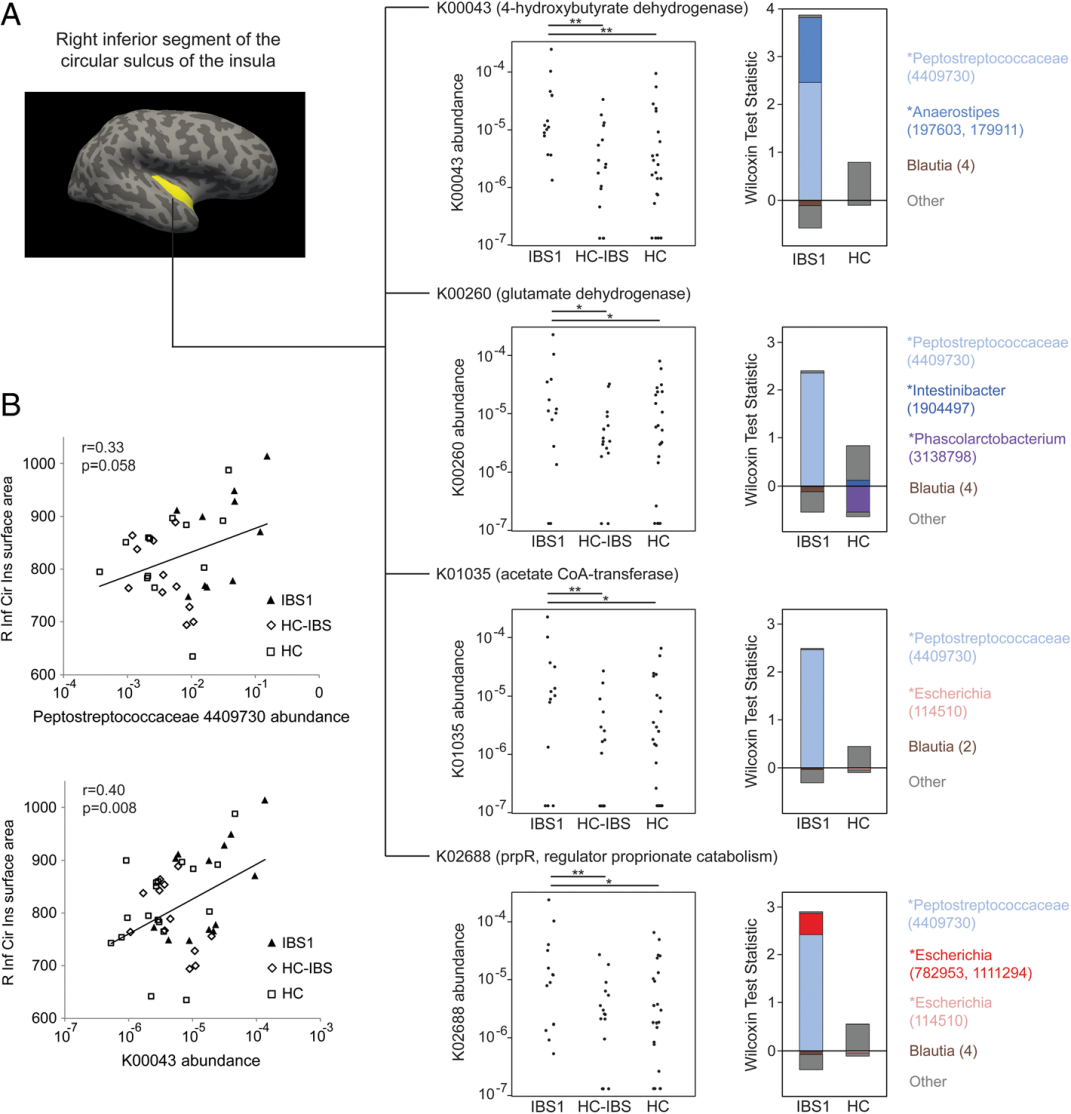
Bacterial genes involved in the metabolism of neurotransmitters and short-chain fatty acids correlate with the surface area of the right inferior segment of the circular sulcus of the insula (R Inf Cir Ins). **a** Relative abundances in IBS1, HC-IBS, and HC are shown for four of the metagenes, e.g., genes predicted by PICRUSt associated with the surface area of the right inferior segment of the circular sulcus of the insula (R Inf Cir Ins). Statistical significance was calculated using the Mann-Whitney *U* test; *p < .05, **p < .01. The contribution of individual taxa to the significance of metagene differential abundance between IBS1 and HC was evaluated using FishTaco. The positive and negative effects of taxa on significance are shown separately for taxa more abundant in IBS1 and those more abundant in HC. Taxa could influence significance either by carrying the predicted gene (marked by an asterisks) or by being correlated with other taxa carrying the gene. “Other” refers to the total effect of all other taxa (out of 363) included in the analysis. **b** Surface area of the R Inf Cir Ins is plotted against the relative abundances of K00043 and the *Peptostreptococcaceae* OTU accounting for the majority of the predicted differential abundance of this metagene. Linear regression trendlines are shown along with the Pearson’s correlation coefficient and *p* value

We investigated on an exploratory basis whether the distinct microbial composition of IBS1 compared to HC-IBS and HC was associated with a shift in the functional profile of the microbiome. This was motivated by the possibility that altered bacterial production of neuroactive metabolites could contribute to the association of microbial clusters with brain morphometric parameters. Counts of bacterial genes (metagenes) were predicted from the 16S rRNA gene sequence data using PICRUSt, which imputes gene content for each OTU based upon the phylogenetically closest reference genome. The resulting count data were fitted to negative binomial models using DESeq2 with microbial cluster as a predictor. In pairwise comparisons, 606 metagenes (i.e., bacterial genes predicted by PICRUSt) differed in abundance between the IBS1 subgroup and HC-IBS with adjusted *p* value <.05 and 1223 metagenes differed between IBS1 and HC. In contrast, there were no differential metagenes between HC-IBS and HC. In parallel analyses, the same methodology was used to identify metagenes associated with the 10 brain morphometric parameters (each analyzed separately) that differed among the three microbial clusters at *p* < .01 (Table 3). Twenty-seven metagenes were found that were significantly associated with both the IBS1 subgroup and with brain morphometric parameters, including 20 that were associated with the surface area of the right inferior segment of the inferior segment of the circular sulcus (a region of the posterior insula) (Additional file 11: Table S7). Of these, we focused on two metagenes enriched in IBS1 that are involved in neurotransmitter metabolism—4-hydroxybutyrate dehydrogenase, which degrades γ-hydroxybutyric acid (GHB), and glutamate dehydrogenase, which mediates glutamate synthesis/breakdown—and on two metagenes enriched in IBS1 that are involved in short chain fatty acid metabolism—acetate co-A transferase subunit B, which mediates the final step of butyrate synthesis, and propionate catabolism operon regulatory protein, which regulates an operon of propionate catabolism genes (Fig. 5a). The individual taxa that made the greatest contribution to the association of these metagenes with IBS1 were identified using FishTaco. This highlighted a prominent role for one particular taxon, an unclassified *Peptostreptococcaceae*, which had a trend toward significant correlation (*p* = .058) with the right inferior segment of the circular sulcus though it fell short of significance as compared to the metagene encoding 4-hydroxybutyrate dehydrogenase (*p* = .008) (Fig. 5b).

## Discussion

This exploratory study aimed to identify possible correlations between gut microbial community structure and brain architecture. The main findings of the study were (1) The identification of IBS subgroups based on distinct microbial clusters with only one of these subgroups (IBS1) showing gut microbial and some behavioral and clinical differences; (2) The identification of differences in the abundance of certain microbial taxa between IBS1 and HCs; and (3) The correlation of microbial taxa and imputed metagenes with structural brain alterations primarily in sensory integration and salience network regions in IBS1. To our knowledge, this is the first report demonstrating a correlation between the architectures of the brain and the gut microbiota in a distinct subgroup of IBS patients. These findings suggest a possible influence of the gut microbiota and their metabolites on specific brain structures which may play a role in the pathophysiology of altered sensory processing in IBS.

### Identification of IBS subgroups, based on microbiome diversity and relative abundance of the phyla *Firmicutes* and *Bacteroidetes*

Both hierarchical clustering using average linkage and PCoA analysis on unweighted Unifrac distances indicated that microbial signatures can be used to identify two subgroups among the IBS subjects, one that has a microbial composition similar to HCs, and one that shows a distinct gut microbial signature. This differentiation was also supported by our Random Forest classifier. Identification of the subgroups was not associated with sequencing depth. As a group, all IBS subjects showed significantly greater alpha diversity and richness than HCs, a difference that was largely explained by the greater diversity within the IBS1 subgroup. Higher alpha diversity has previously been reported in an IBS subgroup [10], in patients with celiac disease [51], and with autism spectrum disorder [52], all syndromes which are often accompanied by IBS-like symptoms. Even though an increased microbial diversity has been associated with diets high in fruits, vegetables, and fiber [53], a detailed dietary analysis between the groups did not reveal significant differences in dietary intake.

Subjects within the IBS1, but not the HC-like group, differed from the HC group in the relative abundances of the phyla *Firmicutes* and *Bacteroidetes* (F-B ratio). While the abundance of *Firmicutes* was significantly greater in IBS1, that of *Bacteroidetes* was lower. In contrast, the HC-like IBS group did not differ from the HC with respect to these relative abundances. The finding of gut microbiota-based IBS subgroups and some of their gut microbial composition is similar to findings recently reported by Jeffery et al*.* [10], even though they identified two IBS clusters that differed from HCs in addition to a subgroup similar to HC. The finding of IBS subgroups based on gut microbial composition that had identical clinical symptoms suggests that the difference in microbial architecture is either not necessary for symptom generation in IBS, or that identical clinical presentations can be caused by different underlying mechanisms, one involving gut microbial alterations. Similar to the current finding in the IBS1 subgroup, both clusters in Jeffrey’s study showed a greater abundance of *Firmicutes* and a lower abundance of *Bacteroidetes*. Altered F-B ratios have been reported in other chronic diseases [54], with increased F-B ratios reported in both preclinical and clinical studies of obesity, metabolic syndrome, and high fat intake [55–57].

The reason for the increased F-B ratio found in this and in previous studies in IBS subjects [8, 9] remains unknown. F-B ratios in healthy individuals are highly variable, despite similarities in microbial function [58]. However, age [59] and diet [55, 57, 60] are important modulatory factors. For example, animal models of obesity, obese individuals, those with metabolic syndrome, and those on a Western diet with high animal fat content were found to have increased F-B ratios [55–57]. Fatty acids in high fat diets, such as in the typical North American diet, have been shown to increase the F-B ratio, and this increase has been associated with an increase in gut epithelial permeability and low grade inflammation [61]. It is intriguing to speculate that the increased F-B ratio in a subgroup of IBS patients is related to alterations in epithelial permeability and low grade inflammation which have been implicated as possible disease mechanisms in IBS [62]. Even though we observed trends for higher BMI and higher plant-derived fat intake in the IBS1 group, these differences did not reach statistical significance. Specific dietary habits, such as increased consumption of fat, have not been reported for IBS [63, 64]. No other dietary differences between the groups were identified.

In addition to the differences in relative abundances at the phylum level, several differences at lower taxonomic levels were also observed. The IBS1 group showed a greater relative abundance of several *Firmicutes*-related taxa, including members of *Bacilli* and *Clostridia* at the class level, of *Bacilli*-associated *Lactobacillales* at the order level, and of *Holdemania* at the genus level. The IBS-associated enrichment in *Clostridia* is particularly interesting in light of the finding that select spore-forming bacteria, dominated by *Clostridia* Cluster IV and XIVa, sufficiently induce serotonin biosynthesis by colonic enterochromaffin cells [65]. This aligns well with the reported links between intestinal serotonin dysregulation and IBS [66]. *Holdemania* are commonly found in the healthy gut, but there are few generalizable results for this genus. IBS1 differed in these relative abundances from both HC-like IBS and HCs, with the exception of differences in the abundance of *Lactobacillales*, which were only seen in comparison to HCs. This finding is surprising in view of the common recommendation of probiotics to treat IBS symptoms, the majority of which contain *Lactobacilli*. As acute laboratory stressors have been shown to decrease *Lactobacilli* in the stool in both clinical and preclinical studies, one would expect a reduction of *Lactobacilli* in a stress-sensitive disorder like IBS.

In addition to the increased abundance in some microbes, the IBS1 group had lower relative abundances of *Bacteriodia* at the class level, of *Bacteroidales* at the order level, and of *Parabacteriodes* at the genus level. All these differences were significant when compared to the HC group, but not the HC-like IBS group. The random forest analysis showed that OTUs contributing to the differentiation of IBS1 from HC gut communities included members of the genera *Blautia*, *Streptococcus*, *Faecalibacterium*, and *Bacteroides*.

The reasons that the group differences in relative abundance at lower taxonomic levels differed from those reported by others [9, 10, 67] are unknown, but may include differences in patient populations, diet, DNA extraction techniques, and the primers used for amplicon generation, as well as differences with respect to bioinformatic pipelines, data transformations, and the statistical approaches applied to the data.

### Correlation of gut microbial composition with behavioral and clinical parameters

Similar to Jeffrey’s findings, we found few correlations between the gut microbial-based subgroups and clinical parameters such as IBS symptom severity, predominant bowel habit, or medication use, except for a moderate correlation with symptom duration. As subjective ratings of predominant bowel habits generally show poor correlations with colonic transit times, and the colonic transit times are normal in the majority of IBS patients regardless of reported predominant bowel habit, the observed lack of correlation of such ratings with microbial composition is not surprising. As commonly observed in clinical studies, IBS patients as a group had a significantly higher level of anxiety symptoms while IBS1 and HC-like IBS did not differ from each other. However, no correlations between anxiety (or depression) symptom scores and microbial parameters were observed.

Interestingly, IBS1 had significantly greater scores on the emotional scale of the ETI than both the HC-like IBS and HCs. While the relative abundance of the *Firmicutes*-associated class *Bacilli* was positively correlated with ETI total score, as well as with scores in the sexual and emotional subscales of the ETI, no correlations with EAL were found for the *Firmicutes*-associated class *Clostridia*, or the *Bacteriodetes*-associated class *Bacteroidia*. Even though the observed correlation between a history of ELAs and the microbiome needs to be confirmed in future studies, one may speculate that brain driven disturbances of the gut microbial environment in early life [12] may have a long lasting effect on gut microbial composition persisting throughout life, which in turn may lead to further changes in brain structure/function.

### Correlation with brain structures

Moderate-sized correlations with brain structure were observed for certain *Firmicutes*- and *Bacteroides*-associated taxa. For example, the *Firmicutes*-associated *Clostridia* (higher in IBS1) and the *Bacteroidetes*-associated *Bacteroidia* (lower in IBS1) showed correlations with the volume of several subcortical brain regions involved in sensory integration and modulation and the motor cortex. For the majority of these regions, *increased* volumes were observed with decreases in *Bacteroidia* taxa and increases in the *Clostridia* taxa characterizing IBS1. On the other, decreased volumes of the anterior insula and ventral prefrontal regions were associated with the taxa profile of IBS1. Whether *Clostridia*-mediated modulation of peripheral serotonin levels [65] may be involved is unclear, increasing evidence reveals microbiome-mediated changes in neurochemical signaling and neurophysiology. The abundance of the *Firmicutes*-associated *Bacilli* (increased in IBS1) was related to only a few regional brain volumes including positive correlations with the right nucleus accumbens and subregions of the frontal gyrus, and negative correlations with other prefrontal cortices and the posterior cingulate cortex.

Previous structural and white matter studies have shown IBS-related alterations in some of the same regions, with IBS patients showing larger gray matter volumes [68] and altered white matter tracts in the thalamus and basal ganglia [69], and reduced gray matter volumes in insula and prefrontal cortices [68]. Several possible explanations for these structural brain changes have been proposed, including genetic and epigenetic factors, the effect of gut microbial metabolites, as well as the effect of longstanding increased sensory signaling from the periphery [6].

The biological mechanisms underlying the observed correlations remain to be determined. For the sensory brain regions, it is conceivable that neuroactive or proinflammatory metabolites generated by altered gut microbiota reach the brain, inducing neuroplastic changes. As most patients with IBS symptoms have a longstanding history of symptoms, often dating back to childhood, it is likely that such altered gut microbiota to brain signaling could have shaped the brain from early on in life. This view is also consistent with the observed correlation of microbial composition with EALs.

In support of this possibility, we found that the surface area of the posterior insula was associated with the predicted abundance of 20 bacterial genes increased in the IBS1 group. The posterior insula is considered the primary visceral cortex and was chosen a priori without corrections for type I error. The identified genes included two that influence synthesis/degradation of GHB and glutamate. GHB is a neurotransmitter found naturally at high levels in the intestine that inhibits intestinal peristalsis via GABA_B_ receptors and has sedative effects in the CNS [70, 71]. Glutamate is an excitatory neurotransmitter in the enteric nervous system and in the brain where it also plays an important role in synaptic plasticity [72]. The posterior insula was also associated with abundance of a subunit of butyryl-CoA:acetate CoA-transferase, an enzyme used by intestinal bacteria such as *Faecalibacterium* in the final step of butyrate synthesis [73, 74]. Butyrate has histone deacetylase activity and signals through GRP43 and GRP109a (expressed by enterochromaffin cells, vagal afferents, and microglia), all potential mechanisms by which it could influence brain function or structure [75]. Interestingly, a positive regulator of proprionate catabolism operon was also associated with this region of the posterior insular, suggesting a shift in the short-chain fatty acid profile [76]. Association of these metagenes with IBS1 group was largely attributable to a single unclassified member of the *Peptostreptococcaceae*. It is unclear why this taxon bloomed in a subset of IBS patients, though diet or fiber supplementation are possibilities [77]. Mechanistic studies in rodent models are warranted to investigate these hypotheses.

There are several limitations to this study. The sample was relatively small and composed of both male and female subjects and did not include measures of intestinal transit times. Self-ratings of bowel habits are known to have a poor correlation with intestinal transit time. All correlations with brain structure were cross sectional, and no conclusion about causality can be made from our results. The observed gut microbial changes could be secondary to altered autonomic nervous system output to the gut, changing the microbial environment [78]. Alternatively, the brain changes could be the consequence of altered signaling to the brain through microbial metabolites, or both mechanisms may be involved [6]. Sequencing depth of the microbiome may be a limitation as rare members of the microbiota may be undetected, and differences in sequencing depth may affect relative distributions of microbial taxa. Finally, predicted metagenomics analysis to impute potential metabolites involved in the observed structural brain differences is limited, and results need to be confirmed by metabolomics analyses.

## Conclusions

In summary, this is to our knowledge the first report demonstrating an association of gut microbial composition and function with regional brain structural changes in IBS. Regardless of the causation underlying the observed associations, several intriguing conclusions can be made from this study. Both the correlations of abundance of certain microbial taxa with early adverse life events and with distinct brain structural changes previously reported in IBS suggest a possible role of gut microbes and their metabolites in the development and shaping of the gut-microbiota-brain axis early in life. Confirming results from a previous study [10], IBS subgroups can be identified based on gut microbial composition, which do not correlate with clinical findings such as bowel habits, or with psychological symptom scores. These findings suggest the possibility of a new classification of IBS patients based on gut microbial signatures (and eventually on metabolomics profiles) rather than on clinical characteristics. Furthermore, consistent with clinical observations, the findings suggest the possibility that treatments aimed at altered gut microbial composition with antibiotics, probiotics, prebiotics, and certain diets may only work in subgroups of patients with an altered gut microbiome. Not only could such subgroups explain the lack of response to such treatments in a significant proportion of patients but also the worsening of IBS symptoms in subsets of patients with intake of food, fiber supplementation, and even probiotics. Identifying IBS subgroups based on gut microbiota, their related metabolomic profiles and corresponding brain signatures is likely to play an important role in optimizing therapies in IBS.

## Additional files

Additional file 1: Table S1. NHANES. (DOCX 13 kb)

Additional file 2: Table S2. Cortical and subcortical brain regions. (DOCX 17 kb)

Additional file 3: Bioinformatics Workflow. (TXT 9.85 kb)

Additional file 4: Figure S1. Two-dimensional plots of the principal coordinate analysis. The plot of principal component (PC) 3 versus PC 2 demonstrates presence of clusters or groupings based upon operational taxonomic unit (OTU)-level microbial features. IBS subjects are represented as blue squares. Healthy control subjects are represented by red circles. (PNG 36 kb)

Additional file 5: Table S3. Relative abundances of observable taxonomic units (OTUs) correlated most strongly with the location along the 3rd axis of the principal coordinate analysis (*r* > .45). (DOCX 14 kb)

Additional file 6: Figure S2. Faith’s phylogenetic alpha diversity curves depicting richness of operational taxonomic units (OTUs) as a function of sequencing depth. Rarefaction curves are a plot of the number of species as a function of the number of samples. (PNG 476 kb)

Additional file 7: Table S4. OTUs contributing to the differentiation of IBS versus HC gut communities. (DOCX 16 kb)

Additional file 8: Figure S3. Receiver-operating characteristic (ROC) curve of the operational taxonomic unit (OTU)-based Random Forest model which correctly distinguished the IBS1 subtype from HC and HC-IBS. AUROC: area under the ROC curve. (PNG 2134 kb)

Additional file 9: Table S5. Relative abundance of operational taxonomic units and taxa showing significant group differences. (DOCX 20 kb)

Additional file 10: Table S6. Mean difference in clinical metadata between IBS subgroups based on microbiota IBS subgroups. (DOCX 21 kb)

Additional file 11: Table S7. Metagene associations with brain morphometry and microbiota clusters. (DOCX 18 kb)
