# Supplementary material for: Differences in gut microbial composition correlate with regional brain volumes in irritable bowel syndrome
Source: Microbiome. 2017 May 1;5:49. doi: 10.1186/s40168-017-0260-z (PMC5410709; doi:10.1186/s40168-017-0260-z)
Supplement: Supplementary file 1 — NHANES. (DOCX 13 kb) [file 40168_2017_260_MOESM1_ESM.docx]

Table S1. NHANES

*Food grouping for use in dietary analysis*

| Food Group | | Food and food groups from the NHANES III food-frequency questionnaire |
| --- | --- | --- |
| Animal fat sources | Beef (hamburger, steaks roast beef, and meatloaf), pork (roast pork, pork chops, spare rib, ham, and bacon) liver, sausage, poultry, luncheon meats, milk, yogurt, cottage cheese, cheese, ice cream, eggs, and butter | |
