# Supplementary material for: Differences in gut microbial composition correlate with regional brain volumes in irritable bowel syndrome
Source: Microbiome. 2017 May 1;5:49. doi: 10.1186/s40168-017-0260-z (PMC5410709; doi:10.1186/s40168-017-0260-z)
Supplement: Supplementary file 2 — Cortical and subcortical brain regions. (DOCX 17 kb) [file 40168_2017_260_MOESM2_ESM.docx]

**Table S2. Cortical and Subcortical Brain Regions**

| **Destrieux Atlas Regions** | **ShortName** | **Region** |
| --- | --- | --- |
| Anterior part of thecingulate gyrus and sulcus(ACC) | ACgG_S | Cingulate |
| Middle-anterior part of thecingulate gyrus and sulcus(aMCC) | MACgG_S | Cingulate |
| Middle-posterior part of thecingulate gyrus and sulcus(pMCC) | MPosCgG_S | Cingulate |
| Posterior-dorsal part of thecingulate gyrus(dPCC) | PosDCgG | Cingulate |
| Posterior-ventral part of the cingulate gyrus (vPCC,isthmus of the cingulate gyrus) | PosVCgG | Cingulate |
| Subcallosal area, subcallosal gyrus | SbCaG | Cingulate |
| Marginal branch(or part)of the cingulate sulcus | CgSMarp | Cingulate |
| Pericallosal sulcus (S of corpus callosum) | PerCaS | Cingulate |
| Fronto-marginal gyrus (of Wernicke) and sulcus | FMarG_S | Frontal |
| Paracentral lobule and sulcus | PaCL_S | Frontal |
| Subcentral gyrus (central operculum) and sulci | SbCG_S | Frontal |
| Transverse frontopolar gyri and sulci | TrFPoG_S | Frontal |
| Opercular part of the inferior frontal gyrus | InfFGOpp | Frontal |
| Orbital part of the inferior frontal gyrus | InfFGOrp | Frontal |
| Triangular part of the inferior frontal gyrus | InfFGTrip | Frontal |
| Middle frontal gyrus(F2) | MFG | Frontal |
| Superior frontal gyrus(F1) | SupFG | Frontal |
| Orbital gyri | OrG | Frontal |
| Precentral gyrus | PRCG | Frontal |
| Straight gyrus, Gyrus rectus | RG | Frontal |
| Inferior frontal sulcus | InfFS | Frontal |
| Middle frontal sulcus | MFS | Frontal |
| Superior frontal sulcus | SupFS | Frontal |
| Lateral orbital sulcus | LORs | Frontal |
| Medial orbital sulcus (olfactory sulcus) | MedOrS | Frontal |
| Orbital sulci(H-shaped sulci) | OrS | Frontal |
| Parieto-occipital sulcus(orfissure) | POcS | Frontal |
| Postcentral sulcus | PosCS | Frontal |
| Suborbital sulcus (sulcus rostrales, supraorbital sulcus) | SbOrS | Frontal |
| Long insular gyrus and central sulcus of the insula | LoInG_CInS | Insular |
| Short insular gyri | ShoInG | Insular |
| Anterior segment of thecircular sulcus of the insula | ACirIns | Insular |
| Inferior segment of thecircular sulcus of the insula | InfCirIns | Insular |
| Superior segment of thecircular sulcus of the insula | SupCirInS | Insular |
| Horizontal ramus of theanterior segment of the lateral sulcus(orfissure) | ALSHorp | Insular |
| Vertical ramus of theanterior segment of the lateral sulcus(orfissure) | ALSVerp | Insular |
| Posterior ramus(or segment)of the lateral sulcus(orfissure) | PosLS | Insular |
| Lateral occipito-temporal gyrus(fusiform gyrus, O4-T4) | FuG | Interlobe |
| Lingual gyrus,ligual part of the medial occipito-temporal gyrus, (O5) | LinG | Interlobe |
| Central sulcus(Rolando'sfissure) | CS | Interlobe |
| Anterior transverse collateral sulcus | ATrCoS | Interlobe |
| Posterior transverse collateral sulcus | PosTrCoS | Interlobe |
| Lateral occipito-temporal sulcus | LOcTS | Interlobe |
| Medial occipito-temporal sulcus (collateral sulcus) and lingual sulcus | CoS_LinS | Interlobe |
| Inferior occipital gyrus (O3) and sulcus | InfOcG_S | Occipital |
| Cuneus(O6) | cun | Occipital |
| Middle occipital gyrus (O2, lateral occipital gyrus) | MOcG | Occipital |
| Superior occipital gyrus (O1) | SupOcG | Occipital |
| Occipital pole | OcPo | Occipital |
| Calcarine sulcus | CcS | Occipital |
| Middle occipital sulcus and lunatus sulcus | MOcS_LuS | Occipital |
| Superior occipital sulcus andtransverse occipital sulcus | SupOcS_TrOcS | Occipital |
| Anterior occipital sulcus andpreoccipital notch(temporo-occipital incisure) | AOcS | Occipital |
| Angular gyrus | AngG | Parietal |
| Supramarginal gyrus | SuMarG | Parietal |
| Superior parietal lobule(lateral part of P1) | SupPL | Parietal |
| Postcentral gyrus | PosCG | Parietal |
| Precuneus(medial part of P1) | PrCun | Parietal |
| Sulcus intermedius primus (of Jensen) | JS | Parietal |
| Intraparietal sulcus(interparietal sulcus) and transverse parietal sulci | IntPS_TrPS | Parietal |
| Inferior part of the precentral sulcus | InfPrCS | Frontal |
| Superior part of the precentral sulcus | SupPrCs | Frontal |
| Subparietal sulcus | SbPS | Parietal |
| Parahippocampal gyrus, parahippocampal part of the medial occipito-temporal gyrus,(T5) | PaHipG | Temporal |
| Anterior transverse temporal gyrus(of Heschl) | HG | Temporal |
| Lateral aspect of thesuperior temporal gyrus | SupTGLp | Temporal |
| Planum polare of thesuperior temporal gyrus | PoPl | Temporal |
| Planum temporale or temporal planeof the superior temporal gyrus | TPl | Temporal |
| Inferior temporal gyrus(T3) | InfTG | Temporal |
| Middle temporal gyrus(T2) | MTG | Temporal |
| Temporal pole | Tpo | Temporal |
| Inferior temporal sulcus | InfTS | Temporal |
| Superior temporal sulcus(parallel sulcus) | SupTS | Temporal |
| Transverse temporal sulcus | TrTs | Temporal |
|  |  |  |
| **Harvard-Oxford Atlas Regions** |  |  |
| Left-Cerebellum-Cortex | L_CeB | Subcortical |
| Left-Thalamus-Proper | L_Tha | Subcortical |
| Left-Caudate | L_CaN | Subcortical |
| Left-Putamen | L_Pu | Subcortical |
| Left-Pallidum | L_Pal | Subcortical |
| Brain-Stem | Bstem | Subcortical |
| Left-Hippocampus | L_Hip | Subcortical |
| Left-Amygdala | L_Amg | Subcortical |
| Left-Accumbens-area | L_Nacc | Subcortical |
| Right-Cerebellum-Cortex | R_CeB | Subcortical |
| Right-Thalamus-Proper | R_Tha | Subcortical |
| Right-Caudate | R_CaN | Subcortical |
| Right-Putamen | R_Pu | Subcortical |
