# Supplementary figures and images for: Differences in gut microbial composition correlate with regional brain volumes in irritable bowel syndrome
Source: Microbiome. 2017 May 1;5:49. doi: 10.1186/s40168-017-0260-z (PMC5410709; doi:10.1186/s40168-017-0260-z)

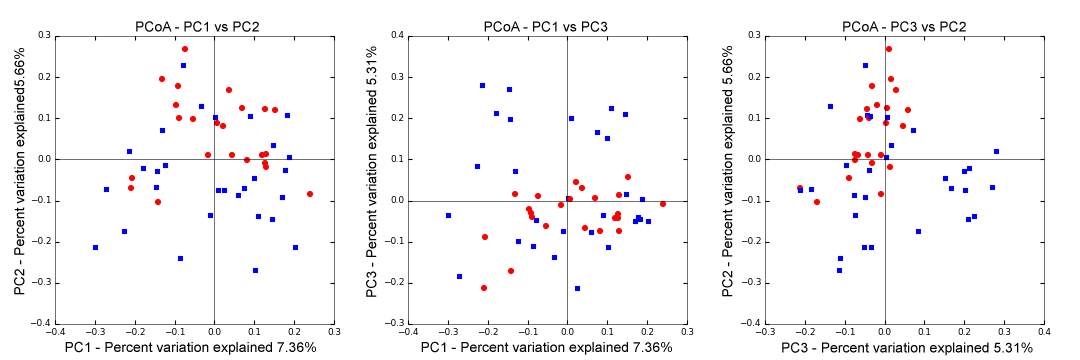

Supplement: Supplementary file 4 — Two-dimensional plots of the principal coordinate analysis. The plot of principal component (PC) 3 versus PC 2 demonstrates presence of clusters or groupings based upon operational taxonomic unit (OTU)-level microbial features. IBS subjects are represented as blue squares. Healthy control subjects are represented by red circles. (PNG 36 kb) [file 40168_2017_260_MOESM4_ESM.png]

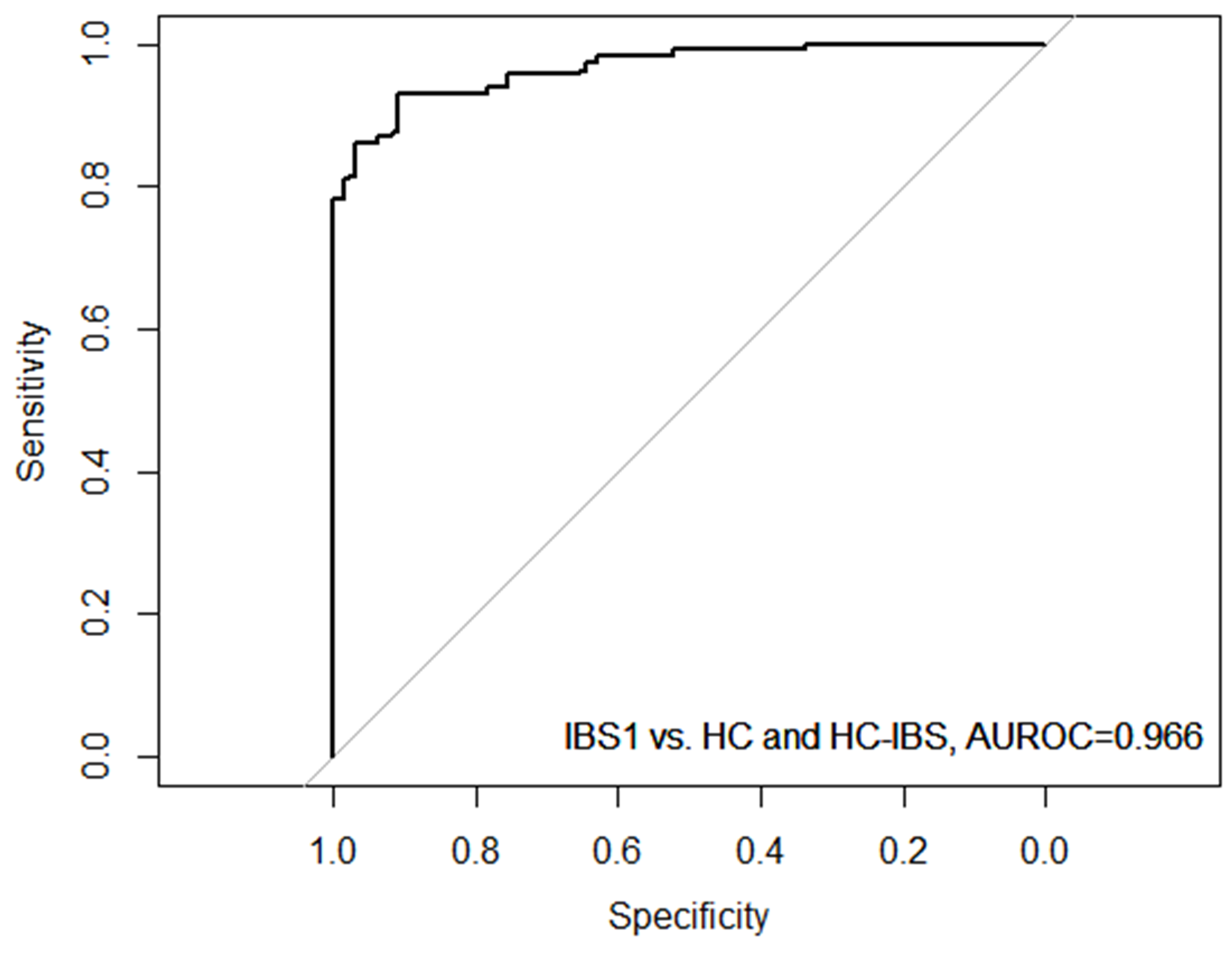

Supplement: Supplementary file 6 — Faith’s phylogenetic alpha diversity curves depicting richness of operational taxonomic units (OTUs) as a function of sequencing depth. Rarefaction curves are a plot of the number of species as a function of the number of samples. (PNG 476 kb) [file 40168_2017_260_MOESM6_ESM.png]

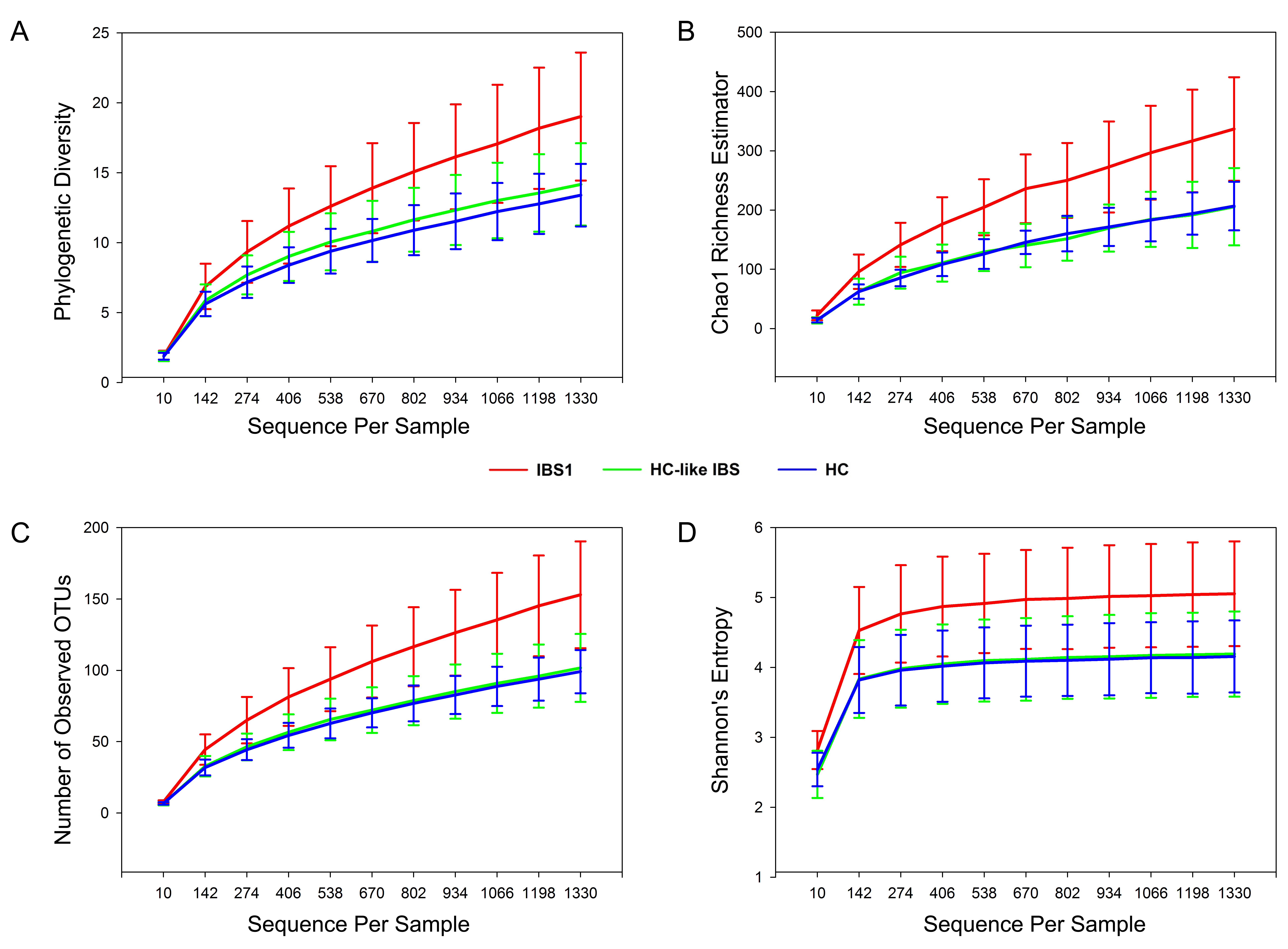

Supplement: Supplementary file 8 — Receiver-operating characteristic (ROC) curve of the operational taxonomic unit (OTU)-based Random Forest model which correctly distinguished the IBS1 subtype from HC and HC-IBS. AUROC: area under the ROC curve. (PNG 2134 kb) [file 40168_2017_260_MOESM8_ESM.png]
