# Supplementary material for: Differences in gut microbial composition correlate with regional brain volumes in irritable bowel syndrome
Source: Microbiome. 2017 May 1;5:49. doi: 10.1186/s40168-017-0260-z (PMC5410709; doi:10.1186/s40168-017-0260-z)
Supplement: Supplementary file 7 — OTUs contributing to the differentiation of IBS versus HC gut communities. (DOCX 16 kb) [file 40168_2017_260_MOESM7_ESM.docx]

| **Table S4. OTUs contributing to the differentiation of IBS versus HC gut communities** | | | | |
| --- | --- | --- | --- | --- |
|  |  |  |  |  |
| **Analysis type** | **OTU identity** | **Random Forest importance score or correlation (Pearson's r)** | **Mean relative abundance (IBS)** | **Mean relative abundance (HC)** |
| Correlation | OTU_2689 k__Bacteria;p__Firmicutes;c__Clostridia;o__Clostridiales;f__Ruminococcaceae;g__Faecalibacterium;s__prausnitzii | 0.747 | 0.412 | 0.006 |
| Correlation | OTU_2637 k__Bacteria;p__Bacteroidetes;c__Bacteroidia;o__Bacteroidales;f__Bacteroidaceae;g__Bacteroides;s__ | 0.647 | 0.327 | 0.008 |
| Correlation | OTU_1208 k__Bacteria;p__Firmicutes;c__Clostridia;o__Clostridiales;f__Lachnospiraceae;g__Blautia | 0.634 | 0.319 | 0.000 |
| Correlation | OTU_2087 k__Bacteria;p__Firmicutes;c__Clostridia;o__Clostridiales;f__Lachnospiraceae | 0.620 | 0.314 | 0.003 |
| Correlation | OTU_1003 k__Bacteria;p__Firmicutes;c__Clostridia;o__Clostridiales;f__Lachnospiraceae;g__;s__ | 0.601 | 0.269 | 0.000 |
| Correlation | OTU_1813 k__Bacteria;p__Firmicutes;c__Clostridia;o__Clostridiales;f__Ruminococcaceae;g__Faecalibacterium;s__prausnitzii | 0.596 | 0.308 | 0.043 |
| Correlation | OTU_825 k__Bacteria;p__Firmicutes;c__Clostridia;o__Clostridiales;f__Lachnospiraceae;g__Blautia;s__ | 0.577 | 0.359 | 0.000 |
| Correlation | OTU_2330 k__Bacteria;p__Bacteroidetes;c__Bacteroidia;o__Bacteroidales;f__Rikenellaceae;g__;s__ | 0.568 | 0.167 | 0.007 |
| Correlation | OTU_533 k__Bacteria;p__Firmicutes;c__Clostridia;o__Clostridiales | 0.564 | 0.192 | 0.000 |
| Correlation | OTU_2011 k__Bacteria;p__Firmicutes;c__Clostridia;o__Clostridiales;f__Lachnospiraceae | 0.562 | 0.385 | 0.000 |
| Correlation | OTU_1822 k__Bacteria;p__Firmicutes;c__Clostridia;o__Clostridiales;f__Ruminococcaceae | 0.558 | 0.192 | 0.000 |
| Correlation | OTU_2078 k__Bacteria;p__Firmicutes;c__Bacilli;o__Lactobacillales;f__Streptococcaceae;g__Streptococcus | 0.532 | 0.185 | 0.000 |
|  |  |  |  |  |
| Random forest | OTU_825 k__Bacteria;p__Firmicutes;c__Clostridia;o__Clostridiales;f__Lachnospiraceae;g__Blautia;s__ | 5.770 | 0.359 | 0.000 |
| Random forest | OTU_2078 k__Bacteria;p__Firmicutes;c__Bacilli;o__Lactobacillales;f__Streptococcaceae;g__Streptococcus | 5.266 | 0.185 | 0.000 |
| Random forest | OTU_2412 k__Bacteria;p__Firmicutes;c__Clostridia;o__Clostridiales;f__Lachnospiraceae;g__Blautia;s__ | 5.173 | 0.144 | 0.001 |
| Random forest | OTU_2689 k__Bacteria;p__Firmicutes;c__Clostridia;o__Clostridiales;f__Ruminococcaceae;g__Faecalibacterium;s__prausnitzii | 5.095 | 0.412 | 0.006 |
| Random forest | OTU_1926 k__Bacteria;p__Firmicutes;c__Clostridia;o__Clostridiales;f__Clostridiaceae | 4.623 | 0.197 | 0.036 |
| Random forest | OTU_2508 k__Bacteria;p__Bacteroidetes;c__Bacteroidia;o__Bacteroidales;f__Bacteroidaceae;g__Bacteroides;s__fragilis | 4.550 | 0.173 | 0.002 |
| Random forest | OTU_2637 k__Bacteria;p__Bacteroidetes;c__Bacteroidia;o__Bacteroidales;f__Bacteroidaceae;g__Bacteroides;s__ | 4.384 | 0.327 | 0.008 |
| Random forest | OTU_2087 k__Bacteria;p__Firmicutes;c__Clostridia;o__Clostridiales;f__Lachnospiraceae | 4.222 | 0.314 | 0.003 |
| Random forest | OTU_2417 k__Bacteria;p__Bacteroidetes;c__Bacteroidia;o__Bacteroidales;f__Bacteroidaceae;g__Bacteroides;s__ovatus | 4.094 | 0.145 | 0.222 |
| Random forest | OTU_1474 k__Bacteria;p__Firmicutes;c__Clostridia;o__Clostridiales;f__Ruminococcaceae;g__Oscillospira;s__ | 3.950 | 0.165 | 0.000 |
| Random forest | OTU_1277 k__Bacteria;p__Firmicutes;c__Clostridia;o__Clostridiales;f__Peptostreptococcaceae;g__;s__ | 3.899 | 0.300 | 0.056 |
| Random forest | OTU_1600 k__Bacteria;p__Firmicutes;c__Clostridia;o__Clostridiales;f__Lachnospiraceae | 3.864 | 0.188 | 0.007 |
| Random forest | OTU_1836 k__Bacteria;p__Firmicutes;c__Clostridia;o__Clostridiales;f__Lachnospiraceae;g__Coprococcus;s__ | 3.817 | 0.169 | 0.045 |
| Random forest | OTU_1471 k__Bacteria;p__Firmicutes;c__Erysipelotrichi;o__Erysipelotrichales;f__Erysipelotrichaceae;g__Holdemania;s__ | 3.792 | 0.167 | 0.000 |
| Random forest | OTU_2575 k__Bacteria;p__Firmicutes;c__Clostridia;o__Clostridiales;f__Ruminococcaceae;g__Faecalibacterium;s__prausnitzii | 3.761 | 0.160 | 0.333 |
