# Supplementary material for: Differences in gut microbial composition correlate with regional brain volumes in irritable bowel syndrome
Source: Microbiome. 2017 May 1;5:49. doi: 10.1186/s40168-017-0260-z (PMC5410709; doi:10.1186/s40168-017-0260-z)
Supplement: Supplementary file 9 — Relative abundance of operational taxonomic units and taxa showing significant group differences. (DOCX 20 kb) [file 40168_2017_260_MOESM9_ESM.docx]

Table S5. Relative abundance of operational taxonomic units and taxa showing significant group differences

|  | **IBS**  **N=13** | **HC-like IBS**  **N=16** | **HC**  **N=23** | **Kruskall Wallis**  **Statistic** | **q** |
| --- | --- | --- | --- | --- | --- |
| ***Operational taxonomic units*** | | | | | |
| Unclassified Holdemania | 0.001 (0.001) | 0.000 (0.000) | 0.000 (0.000) | 23.70 | 0.001 |
| Unclassified Streptococcus | 0.002 (0.004) | 0.0001 (0.0003) | 0.0001 (0.0003) | 17.35 | 0.012 |
| Unclassified Bacteria | 0.010 (0.009) | 0.002 (0.002) | 0.002 (0.001) | 16.75 | 0.012 |
| Unclassified Lachnospiraceae | 0.022 (0.012) | 0.007 (0.007) | 0.007 (0.007) | 15.05 | 0.020 |
| Distasonis | 0.001 (0.001) | 0.017 (0.042) | 0.010 (0.13) | 13.11 | 0.043 |
|  | | | | | |
| ***Phylum*** | | | | | |
| Bacteroidetes | 0.203 (0.162) | 0.367 (0.225) | 0.445 (0.191) | 11.04 | 0.018 |
| Firmicutes | 0.761 (0.762) | 0.625 (0.223) | 0.546 (0.192) | 9.34 | 0.028 |
| Unclassified Bacteria | 0.010 (0.008) | 0.002 (.002) | 0.002 (0.001) | 13.87 | 0.009 |
|  |  |  |  |  |  |
| ***Class*** |  |  |  |  |  |
| Bacilli | 0.021 (0.025) | 0.003 (0.003) | 0.004 (0.007) | 14.67 | 0.008 |
| Bacteroidia | 0.203 (0.162) | 0.367 (0.225) | 0.445 (0.191) | 11.04 | 0.023 |
| Clostridia | 0.715 (0.146) | 0.593 (0.198) | 0.521 (0.169) | 9.13 | 0.035 |
| Unclassified Firmicutes | 0.001 (0.002) | 0.0002 (0.001) | 0.0004 (0.001) | 9.53 | 0.035 |
| Unclassified Bacteria | 0.010 (0.008) | 0.002 (0.002) | 0.002 (0.001) | 13.87 | 0.008 |
|  |  |  |  |  |  |
| ***Order*** |  |  |  |  |  |
| Lactobacillales | 0.020 (0.026) | 0.002 (0.004) | 0.003 (0.005) | 12.55 | 0.024 |
| Bacteroidales | 0.203 (0.162) | 0.367 (0.225) | 0.445 (0.191) | 11.04 | 0.027 |
| Unclassified Clostridia | 0.017 (0.027) | 0.011 (0.016) | 0.004 (0.003) | 11.88 | 0.024 |
| Unclassified Firmicutes | 0.001 (0.001) | 0.0003 (0.001) | 0.0004 (0.001) | 9.53 | 0.046 |
| Unclassified Bacteria | 0.010 (0.008) | 0.002 (0.002) | 0.002 (0.001) | 13.87 | 0.024 |
|  |  |  |  |  |  |
| ***Genus*** |  |  |  |  |  |
| Holdemania | 0.001  (0.001) | 0.000 (0.000) | 0.000 (0.000) | 19.89 | 0.005 |
| Parabacteroides | 0.006  (0.011) | 0.030 (0.048) | 0.026 (0.026) | 11.75 | 0.047 |
| Unclassified Peptostreptococcaceae | 0.001 (0.002) | 4.60xe^-5^  (0.0002) | 0.0001 (0.0002) | 13.47 | 0.030 |
| Unclassified Lachnospiraceae | 0.022 (0.012) | 0.007 (0.007) | 0.007 (0.007) | 15.22 | 0.025 |
| Unclassified Clostridia | 0.017 (0.027) | 0.010 (0.016) | 0.004 (0.0025) | 11.88 | 0.047 |
| Unclassified Bacteria | 0.010 (0.008) | 0.002 (0.002) | 0.002 (0.001) | 13.87 | 0.030 |
|  |  |  |  |  |  |

Values represent group mean relative abundance (standard deviation), q= FDR adjusted p values.
