# Supplementary material for: Differences in gut microbial composition correlate with regional brain volumes in irritable bowel syndrome
Source: Microbiome. 2017 May 1;5:49. doi: 10.1186/s40168-017-0260-z (PMC5410709; doi:10.1186/s40168-017-0260-z)
Supplement: Supplementary file 10 — Mean difference in clinical metadata between IBS subgroups based on microbiota IBS subgroups. (DOCX 21 kb) [file 40168_2017_260_MOESM10_ESM.docx]

Table S6. Mean difference in clinical metadata between IBS subgroups based on microbiota IBS subgroups

|  | **HC-like IBS** |  |  | **IBS1** |  |  |  |  |
| --- | --- | --- | --- | --- | --- | --- | --- | --- |
|  | **M** | **SD** |  | **M** | **SD** |  | t | p |
| Sex (% female) | 75% |  | 16 | 69% |  | 13 |  | .53^†^ |
| Age | 24.50 | 5.42 | 16 | 28.00 | 5.70 | 13 | 1.69 | .10 |
| Body Mass Index | 22.42 | 3.09 | 16 | 25.88 | 5.73 | 13 | 2.08 | .047 |
|  |  |  | | | | | | |
| HAD Anxiety | 6.88 | 5.56 | 16 | 7.92 | 4.75 | 13 | .54 | .60 |
| HAD Depression | 2.69 | 2.50 | 16 | 3.92 | 3.69 | 13 | 1.07 | .29 |
| Perceived Stress Score | 17.06 | 7.88 | 16 | 17.83 | 6.16 | 13 | .28 | .78 |
| Coping Scale Questionnaire | 1.44 | 1.29 | 14 | 1.68 | 1.63 | 13 | .43 | .67 |
|  |  |  | | | | | | |
|  |  | Early Traumatic Inventory (ETI)§ | | | | | | |
| ETI General | 1.79  (0-5) | 1.89 | 14 | 2.23  (0-5) | 1.48 | 13 | .68 | .50 |
| ETI Physical | 1.07  (0-4) | 1.38 | 14 | 1.62  (0-4) | 1.76 | 13 | .90 | .38 |
| ETI Emotional | .15  (0-1) | 0.14 | 14 | 2.14  (0-5) | 2.31 | 13 | 3.56 | .002 |
| ETI Sexual | .29  (0-4) | 1.11 | 14 | .54  (0-5) | 1.07 | 12 | .60 | .55 |
| ETI Total | 3.29  (0-10) | 3.69 | 14 | 6.33  (0-17) | 5.60 | 12 | 1.66 | .11 |
|  |  |  |  |  |  |  |  |  |
|  |  | Bowel Habit | | | | | | |
| BSQ Overall Symptoms | 10.00 | 4.20 | 16 | 8.38 | 4.63 | 13 | -0.99 | .33 |
| BSQ Abdominal Pain | 8.63 | 4.70 | 16 | 7.00 | 4.20 | 13 | -0.97 | .34 |
| BSQ Bloating | 11.69 | 4.38 | 16 | 10.00 | 3.81 | 13 | -1.09 | .28 |
| Duration of Symptoms | 5.67 | 3.79 | 15 | 13.77 | 10.48 | 13 | 2.80 | .01 |
|  |  |  | | | | | | |
| Dietary Plant Fat Intake | 17.47 | 12.71 | 14 | 28.43 | 16.09 | 13 | 1.88 | .07 |
|  |  |  | | | | | | |
| Medication Usage^*^ | 86% |  | 15 | 60% |  | 14 |  | .22**^†^** |

**Abbreviations:** HC, healthy controls; IBS, irritable bowel syndrome; M, mean; SD, standard deviation, t= t statistic from independent t-test, p=probability value.

† P value from fisher’s exact test, § range of scores in parentheses, *percentage taking acceptable medication in each group
