# Supplementary material for: Differences in gut microbial composition correlate with regional brain volumes in irritable bowel syndrome
Source: Microbiome. 2017 May 1;5:49. doi: 10.1186/s40168-017-0260-z (PMC5410709; doi:10.1186/s40168-017-0260-z)
Supplement: Supplementary file 11 — Metagene associations with brain morphometry and microbiota clusters. (DOCX 18 kb) [file 40168_2017_260_MOESM11_ESM.docx]

**Table S7. Metagene associations with brain morphometry and microbiota clusters.**

| **Metagene** | **Description** | **IBS1 vs. HC-IBS** | **IBS1 vs. HC** | **Log2 FC** | **p-value** | **adjusted**  **p-value** |
| --- | --- | --- | --- | --- | --- | --- |
| **Right inferior segment of the circular sulcus of the insula (surface area)** | | | | | | |
| K07311 | putative dimethyl sulfoxide reductase subunit YnfG (DMSO reductase) | 1.53 | 2.09 | 0.0034 | 3.9E-05 | 0.045 |
| K02688 | prpR, transcriptional regulator, propionate catabolism operon regulatory | 1.56 | 2.09 | 0.0033 | 5.8E-05 | 0.045 |
| K06917 | tRNA 2-selenouridine synthase | 1.55 | 1.84 | 0.0032 | 7.2E-05 | 0.045 |
| K00043 | 4-hydroxybutyrate dehydrogenase | 1.79 | 1.89 | 0.0033 | 8.2E-05 | 0.045 |
| K04019 | ethanolamine utilization protein EutA | 1.18 | 1.28 | 0.0035 | 9.1E-05 | 0.045 |
| K00260 | glutamate dehydrogenase | 1.28 | 1.59 | 0.0033 | 9.3E-05 | 0.045 |
| K04026 | ethanolamine utilization protein EutL | 1.15 | 1.31 | 0.0036 | 1.0E-04 | 0.045 |
| K02663 | type IV pilus assembly protein PilN | 1.70 | 2.17 | 0.0032 | 1.0E-04 | 0.045 |
| K05816 | sn-glycerol 3-phosphate transport system ATP-binding protein | 1.57 | 1.95 | 0.0031 | 1.1E-04 | 0.045 |
| K04032 | ethanolamine utilization cobalamin adenosyltransferase | 1.17 | 1.27 | 0.0034 | 1.4E-04 | 0.045 |
| K02466 | glucitol operon activator protein | 1.71 | 1.67 | 0.0034 | 1.5E-04 | 0.045 |
| K06193 | PhnA protein | 1.58 | 1.85 | 0.0031 | 1.6E-04 | 0.045 |
| K07038 |  | 1.63 | 2.06 | 0.0032 | 1.7E-04 | 0.045 |
| K04027 | ethanolamine utilization protein EutM | 1.55 | 1.60 | 0.0030 | 1.7E-04 | 0.045 |
| K05311 | central glycolytic genes regulator | 1.46 | 1.98 | 0.0033 | 1.8E-04 | 0.045 |
| K11200 | PTS system, 2-O-A-mannosyl-D-glycerate-specific IIC component | 1.54 | 1.84 | 0.0028 | 1.9E-04 | 0.045 |
| K05792 | tellurite resistance protein TerA | 1.52 | 1.55 | 0.0030 | 1.9E-04 | 0.045 |
| K03810 | virulence factor | 1.57 | 1.58 | 0.0031 | 2.0E-04 | 0.045 |
| K04030 | ethanolamine utilization protein EutQ | 1.18 | 1.14 | 0.0034 | 2.1E-04 | 0.045 |
| K01035 | acetate CoA-transferase beta subunit | 1.50 | 1.93 | 0.0027 | 2.3E-04 | 0.047 |
| **Right superior part of the precentral sulcus (volume)** | | | | | | |
| K07057 |  | -1.28 | -1.33 | 0.0006 | 9.9E-08 | 2.6E-04 |
| K09124 | hypothetical protein | -1.66 | -1.71 | 0.0006 | 4.4E-07 | 5.7E-04 |
| **Long insular gyrus and central sulcus of the insula (volume)** | | | | | | |
| K01744 | aspartate ammonia-lyase | -0.45 | -0.22 | -0.0011 | 2.9E-05 | 0.034 |
| K02897 | large subunit ribosomal protein L25 | -0.38 | -0.21 | -0.0009 | 1.3E-04 | 0.034 |
| K07130 |  | 0.29 | 0.15 | 0.0007 | 1.5E-04 | 0.034 |
| K08602 | oligoendopeptidase F | 0.39 | 0.28 | 0.0009 | 1.6E-04 | 0.034 |
| **Left superior part of the precentral sulcus (cortical thickness)** | | | | | | |
| K00359 | NADH oxidase | 0.94 | 1.25 | -3.31 | 2.6E-06 | 0.011 |

Note: Log2 FC (fold change) refers to the fold change in metagene abundance associated with a 1 unit increase in surface area, volume, or cortical thickness.
